# Selected synthetic strategies to cyclophanes

**DOI:** 10.3762/bjoc.11.142

**Published:** 2015-07-29

**Authors:** Sambasivarao Kotha, Mukesh Eknath Shirbhate, Gopalkrushna T Waghule

**Affiliations:** 1 Department of Chemistry, Indian Institute of Technology-Bombay, Powai, Mumbai-400 076, India

**Keywords:** addition reactions, coupling reactions, cyclophane, metathesis, muscopyridine, name reactions, natural products

## Abstract

In this review we cover various approaches to meta- and paracyclophanes involving popular reactions. Generally, we have included a strategy where the reaction was used for assembling the cyclophane skeleton for further functionalization. In several instances, after the cyclophane is made several popular reactions are used and these are not covered here. We included various natural products related to cyclophanes. To keep the length of the review at a manageable level the literature related to orthocyclophanes was not included.

## Indroduction

**Figure 1:**
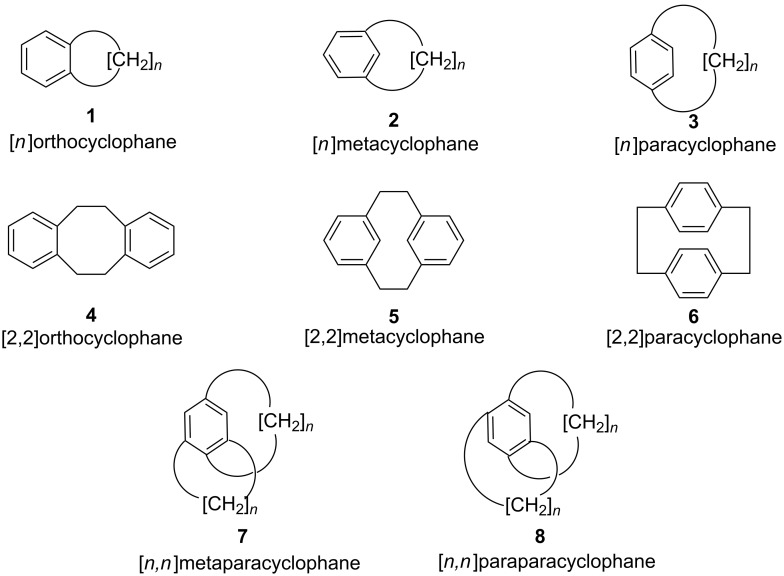
General representation of cyclophanes.

Cyclophanes [1–38] are strained organic molecules which contain aromatic ring(s) as well as aliphatic unit(s). The aromatic rings provide rigidity to their structure, whereas the aliphatic unit(s) form bridge(s) between the aromatic rings and also provide flexibility to the overall structure. Cyclophanes play an important role in “host–guest” chemistry [39–43] and supramolecular assembly [44–47]. “Phane”-containing molecules show interactions with π-systems, and they can also bind to a large number of cations, anions, and neutral molecules. Cyclophanes are widely used in materials science and molecular recognition processes [48–52]. A general classification of cyclophanes is as follows: [*n*]orthocyclophane, [*n*]metacyclophane, and [*n*]paracyclophane (**1–3**) (Figure 1). The prefixes represent the position of the attachment to an aromatic system while [*n*] represents the number of methylene groups present in the aliphatic bridge. The orthocyclophanes are also known as benzocycloalkanes. Several cyclophanes consisting of two or more aromatic systems and aliphatic bridges have been reported in the literature [53]. The representative [2,2]ortho-, meta-, and paracyclophanes (**4**–**6**) are shown in Figure 1. In general, cyclophanes with one aromatic ring and two alkyl bridges are called [*n*,*n*]metapara or [*n*,*n*]paraparacyclophanes (**7**, **8**) based on the position of the attachment of the alkyl chain to the aromatic system. In this review we are not discussing orthocyclophanes but rather focus on meta- and paracyclophanes only.

**Figure 2:**
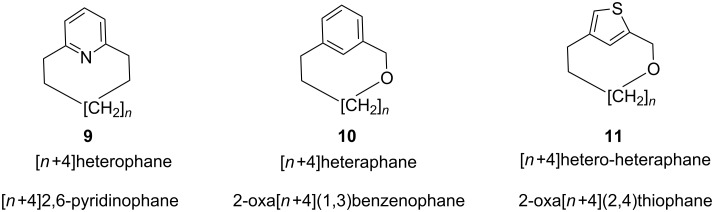
cyclophanes one or more with heteroatom.

The aromatic ring present in the cyclophane system can be either heterocyclic or carbocylic in nature. If there is a heteroatom present in the aromatic ring system then the system is called a heterophane (**9**) [54–56], whereas if the heteroatom is present in the alkyl chain of the bridge, then it is called a heteraphane (**10**) [57–60]. Alternatively, if the heteroatom is present in both the aromatic ring and the alkyl chain, it is called a hetero-heteraphane (**11**, Figure 2).

**Figure 3:**
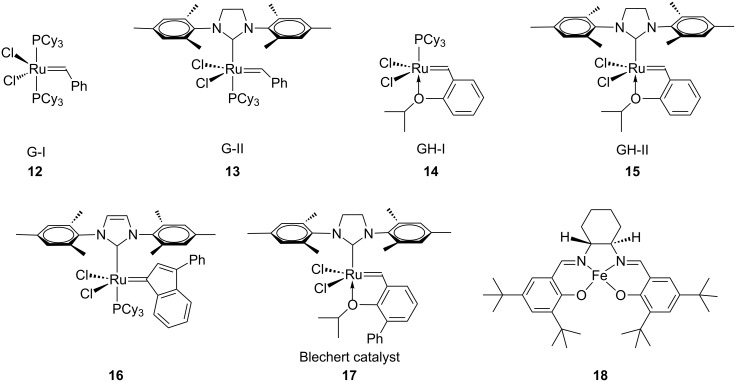
Metathesis catalysts **12–17** and C–C coupling catalyst **18**.

A number of cyclophane derivatives have been employed as hosts, and their guest-binding properties have been widely investigated. A variety of reviews related to the cyclophane chemistry has been published. Although monomeric cyclophanes show moderate guest-binding abilities, an improved affinity can be achieved by polytopic hosts [61–63] through multivalency effects in macrocycles. Olefin metathesis has played a key role in the development of cyclophane chemistry. Some of the catalysts used for this purpose are listed in Figure 3. The development of new synthetic methods in this area is considered a useful exercise. To this end, name reactions or popular reactions, and rearrangement reactions are widely used. In connection with the synthesis of cyclophanes, we describe the employment of these reactions for C–C or C–heteroatom-bond formation. The first part of this review focuses on the syntheses of various cyclophanes related to natural products and the subsequent sections describe the use of various popular reactions in cyclophane synthesis.

### Natural products containing a cyclophane skeleton

The cyclophane skeleton is a core structural unit in many biologically active natural products such as macrocidin A (**19**) and B (**20**) [64], nostocyclyne A (**21**) [65], and in the turriane family of natural products **22–24** [66]. Cyclophanes are also applied in research areas such as pharmaceuticals [67–68], catalysis [69–70] and supramolecular chemistry [71].

**Figure 4:**
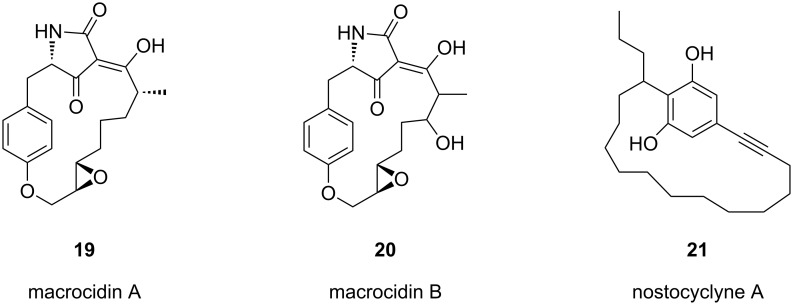
Natural products containing the cyclophane skeleton.

**Figure 5:**
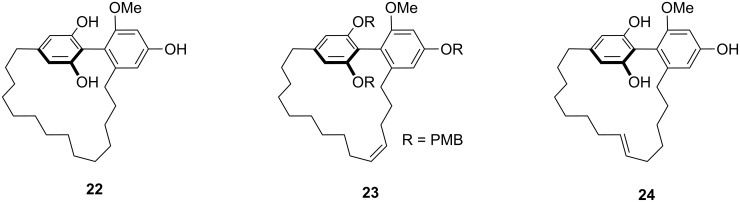
Turriane family of natural products.

Macrocidin A (**19**) and macrocidin B (**20**) [64] belong to a family of plant pathogens produced by *Phoma macrostoma,* a microorganism parasitic to Canadian thistle. Macrocidins contain a tetramic acid group in their skeleton and show selective herbicidal activity on broadleaf weeds but do not affect grasses. Nostocyclyne A (**21**) is an acetylenic cyclophane derivative isolated from a terrestrial *Nostoc* species, with antimicrobial activity (Figure 4). The turriane family of natural products **22–24** were isolated from the stem wood of the Australian tree *Grevillea striata.* Turrianes **22–24** are effective DNA-cleaving agents in the presence of Cu(II). Fürstner and co-workers [72] have reported the total synthesis of natural products **22–24** by using a metathesis reaction [73–82] as the key step. The ring-closing metathesis (RCM) has been utilized for the synthesis of the turriane with a saturated alkyl chain (**22**), whereas the unsaturated turrianes **23**, **24** containing a (*Z*)-alkene moiety have been prepared by alkyne metathesis followed by reduction using Lindlar’s catalyst (Figure 5).

### Muscopyridine and its analogues

Musk is a widly used component in Chinese pharmaceuticals and it has also been used in perfume industry. Muscopyridine was first isolated by a Swiss group [83] from the musk deer (*Moschus moschiferus*). Muscopyridine and its synthetical analogue normuscopyridine are heterophanes, more precisely metapyridinophanes. There are various routes to these compounds and related compounds which are discussed in detail in this review.

## Review

### Synthetic routes to cyclophanes

#### Addition reactions

**Scheme 1:**
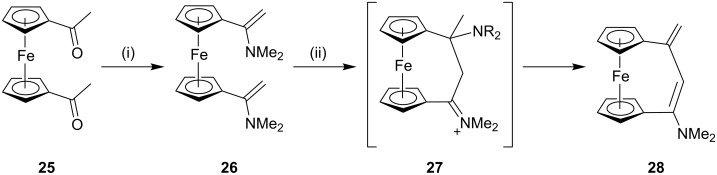
Synthesis of [3]ferrocenophanes through Mannich reaction. Reagents and conditions: (i) excess HNMe_2_; (ii) TiCl_4_, C_5_H_12_, −78 °C, 20 min, 40%.

**Mannich reaction:** In 2001, Erker and co-workers [84] have reported the synthesis of amino-substituted [3]ferrocenophane through an intramolecular Mannich reaction starting with the ferrocene framework. In the first step, the unsaturated amino-functionalized [3]ferrocenophane **28** was synthesized from 1,1’-diacetylferrocene (**25**) in the presence of an excess amount of dimethylamine and a stoichiometric amount of a Lewis acid such as TiCl_4_. These conditions lead to the generation of the bisenamine **26**, which was subsequently converted to the cyclophane **28** by a Mannich-type condensation reaction (40%) (Scheme 1).

**Scheme 2:**
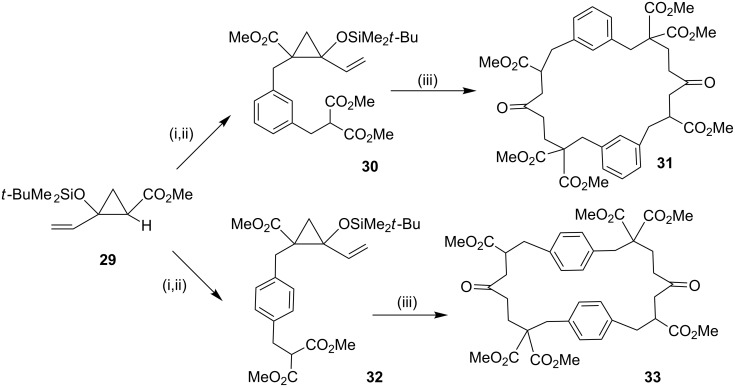
Synthesis of cyclophanes through Michael addition. Reagents and conditions: (i) xylylene dibromide, LDA, −70 °C, 18 h; (ii) NaH, CH_2_(CO_2_Me)_2_; (iii) CsF, BnEt_3_N^+^Cl^−^, DMF, 90 °C, 3 h.

**Michael addition:** In 1999, Reißig and co-workers [85] have synthesized a functionalized cyclophane by a cascade reaction, which proceeds with desilylation, ring opening, proton transfer, and finally, an intramolecular Michael addition to provide benzannulated large ring compounds **31** and **33**. In this regard, substituted methyl 2-alkenyl-2-siloxycyclopropanecarboxylate **29** was converted into the alkylation product and further react with the ester enolate dibromide to yield vinyl cyclopropane derivatives **30** (62%) and **32** (44%). Later, Michael addition in the presence of caesium fluoride and benzyltriethylammonium chloride in DMF gave the benzannulated cyclodecanone derivatives **31** (11%) and **33** (10%) (Scheme 2).

**Scheme 3:**
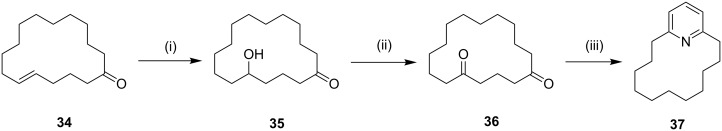
Synthesis of normuscopyridine analogue **37** through an oxymercuration–oxidation strategy. Reagents and conditions: (i) Hg(OAc)_2_, NaSH; (ii) oxidation; (iii) NH_2_OH·HCl.

**Oxymercuration – Hantzsch pyridine synthesis:** Kondo and Miyake [86] have reported the synthesis of [11](2,6)-pyridinophane (**37**), a normuscopyridine analogue, by an oxymercuration–oxidation strategy. The ketoolefin **34** was converted to the hydroxyketone **35** by treatment with Hg(OAc)_2_ and NaSH. Oxidation of the keto alcohol **35** gave diketone **36**, which reacted with hydroxylamine hydrochloride and afforded [11](2,6)-pyridinophane (**37**) (Scheme 3).

#### Coupling reactions

**Scheme 4:**
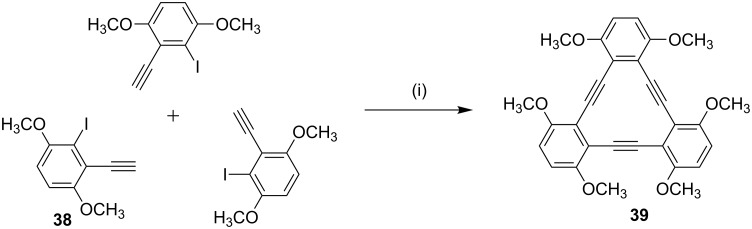
Synthesis of tribenzocyclotriyne **39** through Castro–Stephens coupling reaction. Reagents and conditions: (i) CuCl/ NH_4_OH/EtOH, pyridine, reflux, 24 h, 80%.

**Castro**–**Stephens coupling:** Youngs and co-workers [87] have synthesized *p*-methoxy-substituted tribenzocyclotriyne **39** using the Castro–Stephens coupling reaction (Scheme 4). Compound **39** is a planar antiaromatic dehydroannulene that forms complexes with Ni(0), Cu(I), Co(0) and also with Ag^+^ cations.

**Scheme 5:**
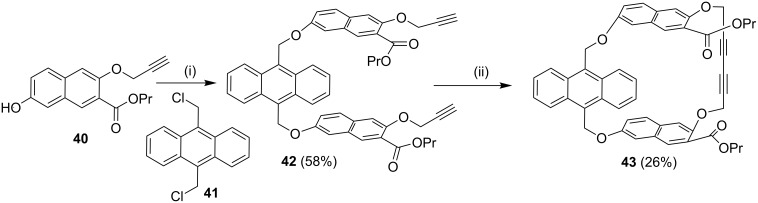
Synthesis of cyclophane **43** through Glaser–Eglinton coupling. Reagents and conditions: (i) 9,10-bis(chloromethyl)anthracene (**41**), Cs_2_CO_3_; (ii) Cu(OAc)_2_·H_2_O, CH_3_CN/pyridine.

**Glaser–Eglinton coupling:** Whitlock and Cloninger [88] have reported the synthesis of cyclophane **43** using the Glaser–Eginton coupling reaction. In this regard, compound **40** was treated with 9,10-bis(chloromethyl)anthracene (**41**) under basic conditions to generate compound **42** which was further subjected to a Glaser–Eglinton coupling to deliver cyclophane **43** (Scheme 5). A derivative of compound **43** was used as a host for compounds such as 6-nitro-2-naphthol, stilbene derivatives and serotonin mimics. This paper depicts the edge–face interaction between the face of the anthracene bridge present in the cyclophane molecule and the edge of the host molecule.

**Scheme 6:**
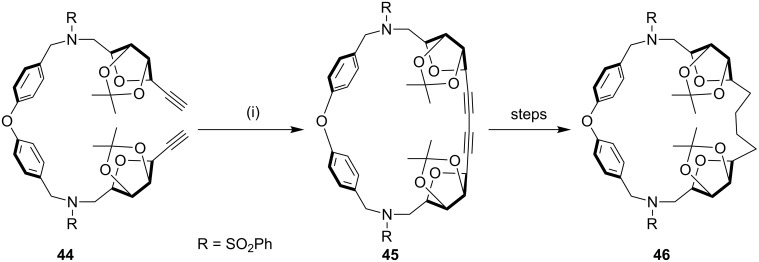
Synthesis of the macrocyclic C-glycosyl cyclophane through Glaser coupling. Reagents and conditions: (i) Cu(OAc)_2_ (10 equiv), pyridine, reflux, 5 h, 54%.

Bukownik and Wilcox [89] have synthesized macrocyclic C-glycosyl compounds, and obtained the chiral and water-soluble cyclophane **46**. They reported on the use of its sulfonamide derivative in preparing glycophane molecule (Scheme 6).

**Scheme 7:**
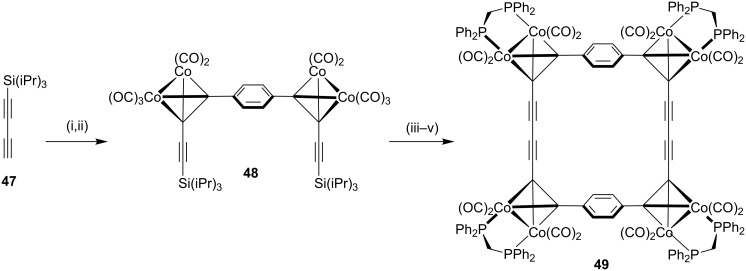
Synthesis of cyclophane-containing complex **49** through Glaser–Eglinton coupling reaction. Reagents and conditions: (i) 1,4-diiodobenzene, Pd(PPh_3_)_2_Cl_2_, CuI, Et_3_N, 25 °C, 24 h, 73%; (ii) Co_2_(CO)_8_, Et_2_O, reflux, 6 h, 66%; (iii) dppm, PhMe, 85%; (iv) Bu_4_NF, THF, >95%; (v) Cu(OAc)_2_·H_2_O, pyridine, reflux, 12 h, 47%.

Haley and Langsdorf [90] have reported the synthesis of a cyclophane-containing octacobalt complex **49** using the Glaser–Eglinton coupling reaction [91] as a key step (Scheme 7). In this regard, palladium-catalyzed alkynylation of 1,4-diiodobenzene with an excess amount of triisopropylsilylbutadiyne (**47**) followed by complexation with Co_2_(CO)_8_ furnished a pale yellow diyne **48**. Exchange of the ligand with bis(diphenylphosphino)methane (dppm) afforded a bridged complex which is stable to fluoride ions. Subsequent desilylation, followed by Glaser–Eglinton coupling of the terminal acetylene groups provided complex **49** in 47% yield as fine, deep maroon crystals.

**Scheme 8:**
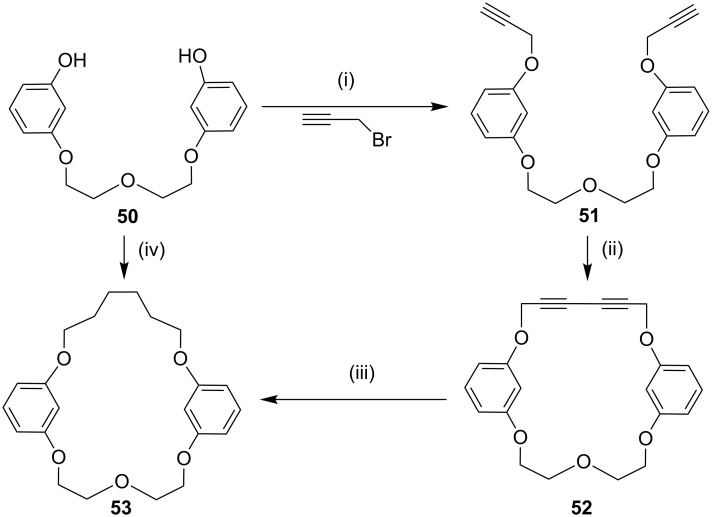
Synthesis of cyclophane **53** through Glaser–Eglinton coupling. Reagents and conditions: (i) K_2_CO_3_, acetone, reflux, 12 h, 86%; (ii) Cu(OAc)_2_·H_2_O, pyridine, CH_3_CN, 60 °C, 2 h, 94%; (iii) H_2_, Pd/C, EtOAc, 12 h, rt, 92%; (iv) 1,6-dibromohexane, K_2_CO_3_, reflux, CH_3_CN, 56%.

In connection with the cyclophane synthesis, Kotha and Waghule [92] demonstrated the use of the Glaser–Eglinton coupling as a key step. The dipropargylated compound **51** was subjected to a Glaser–Eglinton coupling to generate the macrocyclic bisacetylene derivative **52** in 94% yield. Finally, diyne **52** was subjected to a hydrogenation sequence with 10% Pd/C under 1 atm pressure of H_2_ to generate cyclophane derivative **53** (92%). Alternatively, cyclophane **53** was also obtained by treatment of the bisphenol derivative **50** with 1,6-dibromohexane in the presence of K_2_CO_3_ in acetonitrile under reflux conditions (56%, Scheme 8).

**Figure 6:**
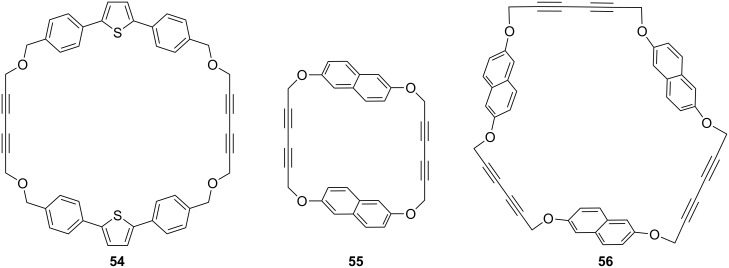
Cyclophanes **54–56** that have been synthesized through Glaser–Eglinton coupling.

Another interesting example of a Glaser–Eglinton coupling reaction reported by Rajakumar and Visalakshi [93] is the synthesis of cyclophane **54**. Whitlock and co-workers have synthesized donut-shaped cyclophanes **55** and **56** by using the Glaser–Eglinton coupling as a key step (Figure 6) [94].

**Figure 7:**
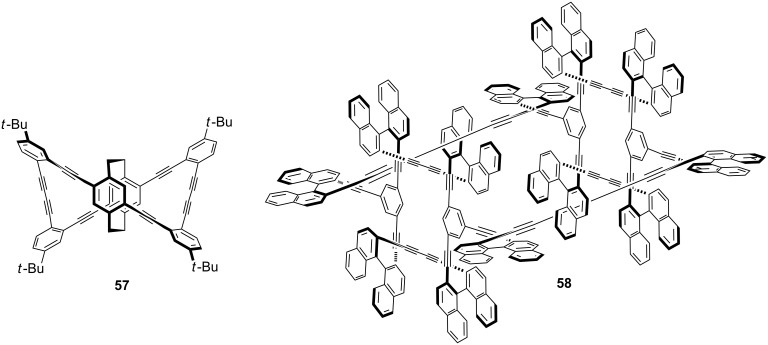
Synthesis of tetrasubstituted [2.2]paracyclophane **57** and chiral cyclophyne **58** through Eglinton coupling.

Morisaki and co-workers [95] have synthesized 4,7,12,15-tetrasubstituted [2.2]paracyclophane **57** and further studies were carried out to find out the properties of these macrocycles. These molecules show excellent chiroptical properties such as high fluorescence quantum efficiency and a large circularly polarized luminescence dissymmetry factor. Cyclophanes are carbon-rich materials containing extensive alkyne moieties with a persistent molecular architecture. Orita and co-workers have reported the synthesis of chiral cyclophyne **58** through the Eglinton coupling reaction [95]. A tandem inter- and intramolecular Eglinton coupling reaction affords the enantiopure three-dimensional cyclophyne **58** with a large cavity size (Figure 7).

**Scheme 9:**
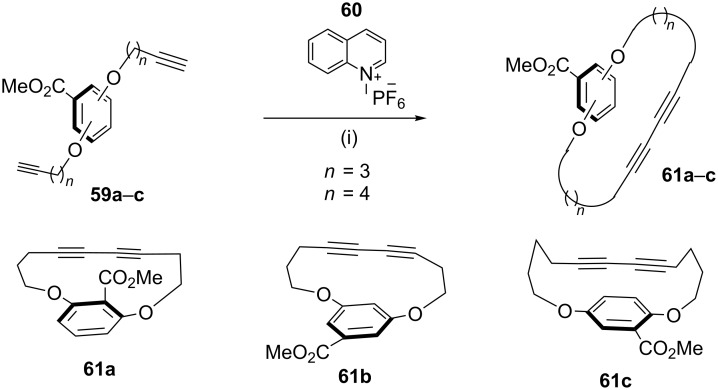
Synthesis of cyclophane through Glaser–Hay coupling reaction. Reagents and conditions: (i) CuCl_2_ (12 equiv), TMEDA (12 equiv), O_2_, PhMe, 18 h, 80 °C.

**Glaser–Hay coupling:** In 2010, Collins and co-workers [96] demonstrated a macrocylization, with an inbuilt conformation control element to form rigid cyclophanes through the Glaser–Hay coupling. In this regard, diynes **59a–c** were treated with CuCl_2_ and TMEDA in the presence of oxygen to afford the cyclized products **61a–c** (Scheme 9).

**Scheme 10:**
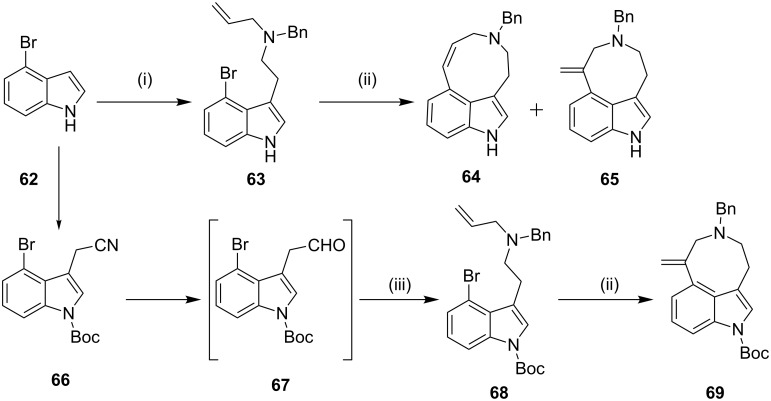
Synthesis of *seco*-C/D ring analogs of ergot alkaloids through intramolecular Heck reaction. Reagents and conditions: (i) (a) POCl_3_, DMF, 0–40 °C, 1 h, 78%; (b) MeNO_2_, cat. NH_4_OAc, reflux, 3 h, 80%; (c) LiAlH_4_, THF, reflux, 4 h, 88%; (d) PhCHO, NaBH(OAc)_3_, CH_2_Cl_2_/THF, rt, 46%; (e) allyl bromide, MeCN, rt, 24 h, 69–72%; (ii) 25 mol % Pd(OAc)_2_, 55 mol % P(*o*-Tol)_3_, NEt_3_, MeCN, reflux, 12 h; (iii) DIBAL-H, 0 °C, 15 min, rt, 2 h, CH_2_Cl_2_, then allylNHMe, NaBH(OAc)_3_, CH_2_Cl_2_, rt, 12 h, 38%.

**Intramolecular Heck coupling:** In 2003, Snieckus and co-workers [97] have synthesized the *seco*-C/D ring analogues of ergot alkaloids through the intramolecular Heck reaction as a key step. The coupling precursors **63** and **68** were prepared from 4-bromoindoles by a sequential Vilsmeier–Haack, Henry nitroaldol condensation, reduction with LiAlH_4_, reductive amination and allylation that afforded the indole derivatives **63** (18%) and *N*-Boc protected compound **68** (23%). The reaction of **63** with Pd(OAc)_2_ (25 mol %) and tri(*o*-tolyl)phosphine (55 mol %) at reflux gave 9-*endo*-**64a** (24%) and 8-*exo*-**65b** (21%). However, the compound **68** under similar reaction conditions gave the cyclized product 8-*exo*-**69** (30%) as the only isolable compound (Scheme 10).

**Scheme 11:**
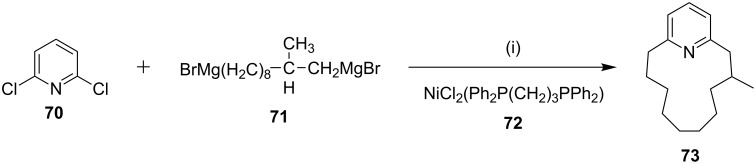
Synthesis of muscopyridine **73** via Kumada coupling. Reagents and conditions: (i) **72**, THF, ether, 20 h, rt, 5–10%.

**Kumada coupling:** Weber and co-workers [98] have synthesized muscopyridine **73** starting from 2,6-disubstituted pyridine. The Kumada cross-coupling reaction of 2,6-dichloropyridine (**70**) with the Grignard reagent **71** in the presence of a nickel phosphine complex **72** gave muscopyridine **73** in a single step (Scheme 11). This strategy has been applied to generate a variety of pyridinophanes by varying the chain length of the Grignard reagent.

**Scheme 12:**
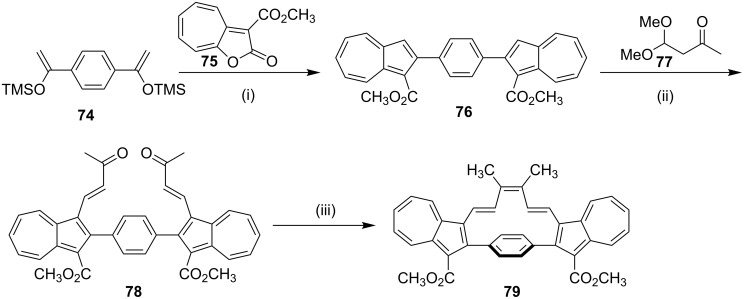
Synthesis of the cyclophane **79** via McMurry coupling. Reagents and conditions: (i) **75**, decaline, reflux, 4 h, 10%; (ii) **77**, NaHCO_3_/HBF_4_, 28%; (iii) TiCl_3_/LiAlH_4_, THF, reflux, 20%.

**McMurry coupling:** Kuroda and co-workers [99] have reported the synthesis of polyunsaturated [10]paracyclophane annulated by two azulene rings by using the McMurry reaction [100–101]. The bis(trimethylsilyl)enol ether **74** was reacted with 3-methoxycarbonyl-2*H*-cyclohepta[*b*]furan-2-one (**75**) in refluxing decaline to generate the 1,4-diazulenobenzene derivative **76**. Double chain elongation of the bis-azulene derivative **76** with a four-carbon unit has been accomplished by electrophilic substitution with 4,4'-dimethoxybutan-2-one (**77**) under acidic conditions and subsequent elimination of methanol under basic conditions gave the advanced precursor **78** (28%). The stereochemistry of the newly generated C–C double bonds in **78** was confirmed as *trans* with the aid of the NMR vicinal coupling constant. Finally, intramolecular McMurry coupling of **78** using titanium trichloride and lithium aluminum hydride (LAH) heated under reflux in THF provided the cyclophane derivative **79** (20%, Scheme 12).

**Scheme 13:**
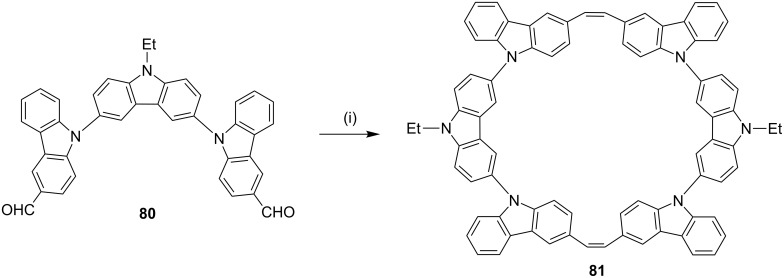
Synthesis of stilbenophane **81** via McMurry coupling. Reagents and conditions: (i) TiCl_4_, Zn, pyridine, THF, reflux, 12 h, 12%.

In another occasion, Rajakumar and co-workers [102] have synthesized a series of stilbenophanes (e.g., **81**) involving N-arylated carbazole moieties possessing small and large cavities. The precursor **80** required for the McMurry reaction was synthesized by the N-arylation of carbazole with the corresponding dibromide followed by formylation (Scheme 13).

**Scheme 14:**
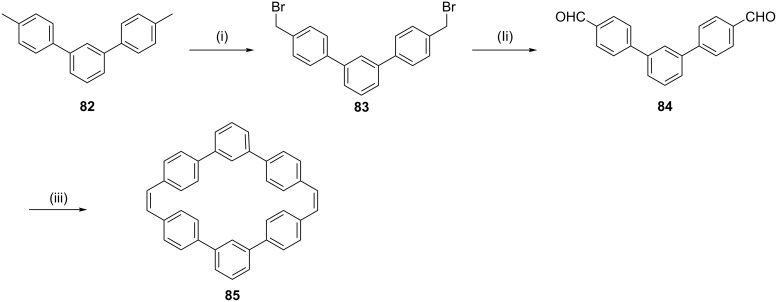
Synthesis of stilbenophane **85** via McMurry coupling. Reagents and conditions: (i) NBS (2 equiv), benzoyl peroxide, CCl_4_, reflux, 40 h, 80%; (ii) tetrabutylammonium dichromate (TBADC), CHCl_3_, reflux, 6 h, 69%; (iii) TiCl_4_ (20 equiv), Zn (40 equiv), pyridine, THF, reflux, 6 h, 28%.

In 2006, Rajkumar and co-workers [103] have published the synthesis of stilbenophane **85** via McMurry coupling as a key step (Scheme 14). Terphenyl derivative **82** was subjected to benzylic bromination in the presence of NBS to generate compound **83**. Later, dibromide **83** was converted to bis-aldehyde **84**. Finally, McMurry coupling of dialdehyde **84** provided the cyclophane derivative **85** (28%).

**Figure 8:**
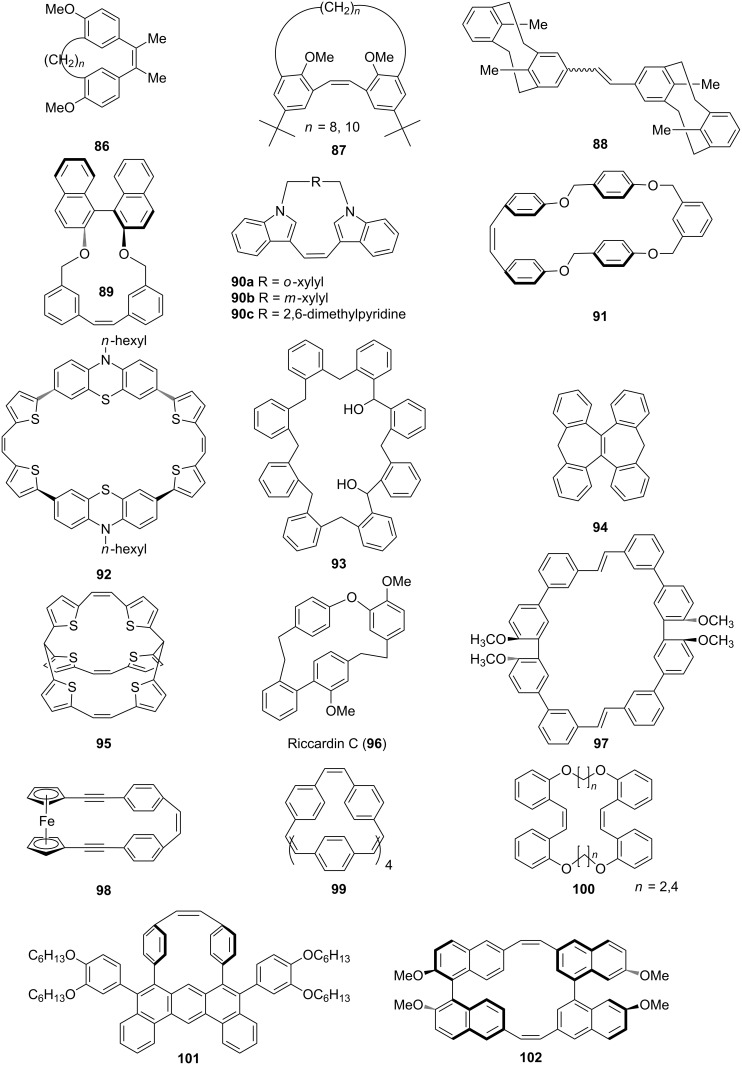
List of cyclophanes prepared via McMurry coupling reaction as a key step.

Yamoto and co-workers have reported the synthesis of medium-sized cyclophanes, [2.*n*]metacyclophane-1,2-diols **86** and **87** by using the McMurry coupling as a key step (Figure 8) [104–106]. Among the π-conjugated systems stilbene derivatives found a unique place in materials science due to their optical and charge conducting properties. Tsuge and co-workers [107] reported the synthesis of stilbene **88** by using the McMurry coupling and studies on the transmission of the electronic effect through transannular interactions. Rajakumar and Selvam [108] also synthesized chiral stilbenophane **89** with small to large cavity sizes. These chiral stilbenophanes forms a complex with tetracyanoethylene (TCNE) and tetracyanoquinodimethane (TCNQ). The same group also reported on the synthesis of indolophanes **90a–c** by using the McMurry coupling [109]. Furthermore, they synthesized dioxastilbenophanes **91** and carried out charge transfer complexation studies which showed that these molecules form a complex with TCNE and TCNQ [110]. Due to the presence of nitrogen and sulfur atoms benzene rings in phenothiaziophanes exhibit a butterfly conformation and thus have shown an enhanced bending character. When the benzene rings are bent, the reactivity of these cyclophanes is altered. Considering this aspect, Müller and co-workers [111] have devised different routes to these molecules. They have reported the synthesis of ethylene-bridged phenothiazinophane **92** using the McMurry coupling reaction. Also cyclic voltammetry experiments indicated the intramolecular electronic communication between the phenothiazinyl subunits. Calixarene-based macrocycles bind with various metal ions. Lee and Park [112] have synthesized various orthocyclophanes **93** which were further converted into spirobicyclic polyketals with a 2*n*-crown-*n*-moiety. Lee and co-workers [113] also reported the synthesis of bicyclic bis-cyclophane **94** by using the McMurry reaction as a key step. Oda and co-workers [114] have reported the first time synthesis of a fully conjugated ionic cyclophane by using the McMurry reaction. The McMurry coupling was carried out with tris(5-formyl-2-thienyl)methane to give an unsubstituted, etheno-bridged trithienylmethanophane **95**. Later, it was converted into the novel cage-molecular monocation, dication, and dianion of substantial stability. Riccardin C (**96**) is a macrocyclic bis-bibenzyl entity with pharmacological properties, including antimycotic and antibacterial effects, and cytotoxicity against P-388 mouse leukaemia and KB cell lines from *nasopharyngeal carcinoma*. In view of these useful medicinal properties Harrowven and co-workers [115] have reported the synthesis of this molecule by using the McMurry reaction. Kawase and co-workers [116] have reported double-helically twisted macrocycles **97** exhibiting chiral sensor properties. Kasahara and co-workers [117] have reported the synthesis of ferrocenophane derivative **98** by McMurry reaction as a key step. Oda and co-workers [118] have reported the synthesis of cyclic paraphenylacetylene in which their spectral properties vary mainly with decrease of ring size of the molecule. They have synthesized intermediate **99** using the McMurry coupling which is required for the synthesis of the paraphenylacetylene compound. Tolanophanes are a new class of cyclophanes possessing a diphenylacetylene moiety which possess interesting structural, electronic, nonlinear optical and luminescent properties. Darabi and co-workers [119] have reported the syntheses of **100** molecules by using the McMurry reaction followed by hydrogenation. Pei and co-workers [120] have synthesized anthracene-based π-conjugated strained cyclophane **101** by using an intramolecular McMurry reaction. The combination of unsaturated linkages in these molecules might create a twisted conformation that imparts helical chirality. Double helically twisted chiral cyclophanes are important macrocycles due to their potential applications in optics and electronics. Kawase and co-workers [121] have reported the synthesis of 8,14,30,36-tetramethoxy[2.0.2.0](1,6)naphthalenophane-1,19-diyne (**102**) using the McMurry coupling (Figure 8).

**Scheme 15:**
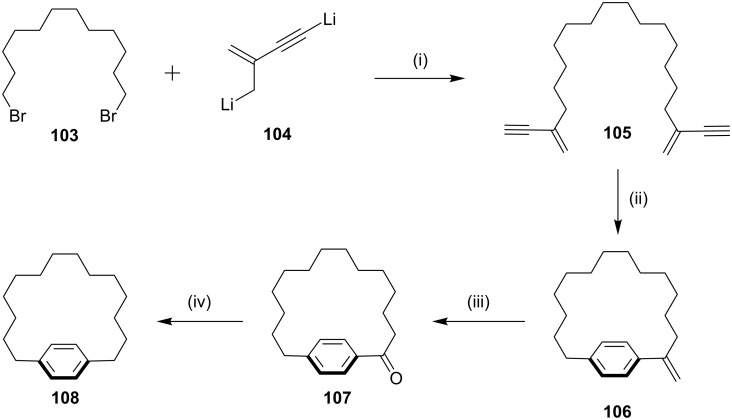
Synthesis of paracyclophane by cross coupling involving Pd(0) catalyst. Reagents and conditions: (i) THF, rt, 72%; (ii) Pd(PPh_3_)_4_, PhMe, high dilution, ∆, 15 min, 32%; (iii) O_3_, −78 °C, Pd/C (10 mol %), H_2_, rt, 55%; (iv) Pd/C (10 mol %), H_2_, 50 °C, rt, 85%.

**Pd(0)-catalyzed cross-coupling reaction:** In 1997, Yamamoto and co-workers [122] have synthesized the exomethylene paracyclophane **108** via intramolecular benzannulation of conjugated enynes in the presence of palladium(0). In this regard, dibromoalkane **103** was treated with dilithiated 2-methyl-1-butene-3-yne (**104**) to generate the corresponding bis-enyne **105**. Treatment with Pd(PPh_3_)_4_ in dry toluene under high dilution conditions at 100 °C afforded the exomethylene paracyclophane **106**. The paracyclophane **106** was converted to oxocyclophane **107** by ozonolysis followed by deoxygenation which finally gave the paracyclophane **108** (85%, Scheme 15).

**Scheme 16:**
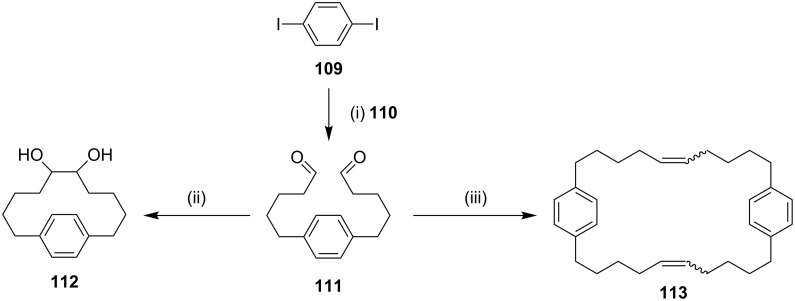
Synthesis of the cyclophane **112** via the pinacol coupling and **113** by RCM. Reagents and conditions: (i) (a) 4-pentyne-1-ol (**110**), CuI, Pd(PPh_3_)_4_, CH_2_Cl_2_/Et_3_N 1:3, 50 °C, 1 d; (b) H_2_, Pd/C, MeOH, rt, 12 h, (two steps 96%); (c) PCC, MS 4 Å, CH_2_Cl_2_, rt, 2 h, 85%; (ii) SmI_2_, HMPA, Mg, TMSCl, rt, 3d, 64%; (iii) (a) H_2_C=PPh_3_, THF, −78 °C to rt, 1 h, 95%; (b) Grubbs first generation catalyst (G-I, **12**), CH_2_Cl_2_, 50 °C, 14 h, 76%.

**Pinacol coupling:** Kanomata and co-workers [123] have reported the synthesis of the cyclophane **112** by using pinacol coupling [124] mediated by SmI_2_. A double Sonogashira reaction of 1,4-diiodobenzene (**109**) with 4-pentyn-1-ol (**110**) generates the diyne product in quantitative yield. Next, the in situ prepared diyne was subjected to hydrogenation followed by oxidation with PCC which gave the dialdehyde **111** (85%). The pinacol coupling of the dialdehyde **111** in the presence of Sm^2+^ and HMPA generated the cyclophane **112** in a moderate yield. RCM of the diene derived from the dialdehyde **111** afforded the macrocyclic cyclophane **113** as a less strained product (Scheme 16).

**Scheme 17:**
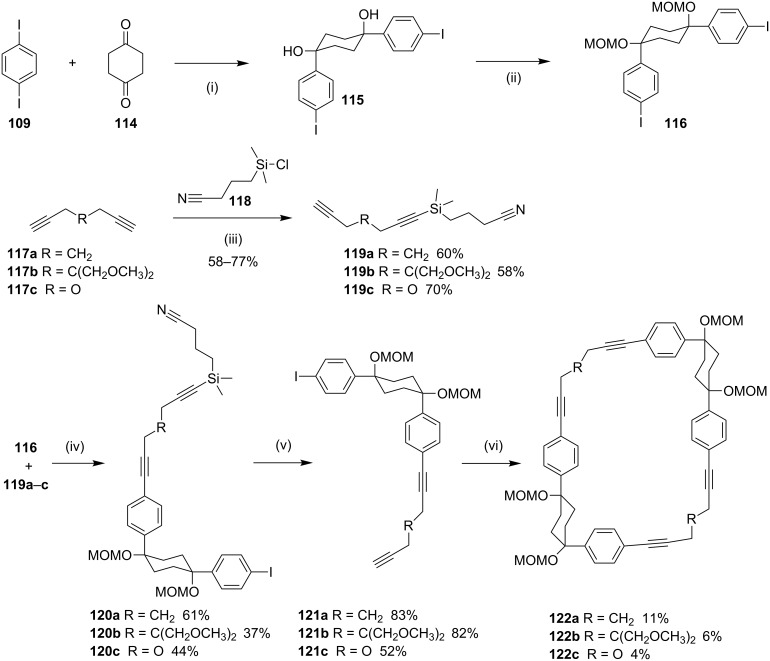
Synthesis of cyclophane derivatives **122a–c** via Sonogoshira coupling. Reagents and conditions: (i) CeCl_3_, LiCl, *n*-BuLi, THF, −78 °C to rt, 2 h, 48%; (ii) MOMCl, iPrNEt_3_, 19 h, rt, 98%; (iii) **118**, EtMgBr, Et_2_O, 30 min, 0 °C then 1 h, rt, further 24 h stirring; (iv) CuI, Pd(PPh_3_)_4_, diisopropylamine, THF, rt; (v) TBAF, THF, 30 min, rt; (vi) CuI (20 mol %), Pd(PPh_3_)_4_ (10 mol %), diisopropylamine, THF, 12 h, rt.

**Sonogashira coupling:** Wegner and co-workers [125] have reported the synthesis of cyclophanes **122a–c** via Sonogoshira coupling [126] (Scheme 17). To this end, the 1,4-diiodobenzene (**109**) was reacted with the cyclohexane-1,4-dione (**114**) in the presence of CeCl_3_/LiCl/*n*-BuLi to generate the diol **115**. Then, the hydroxy groups were protected as MOM groups to generate the key synthone **116**. The other building blocks **119a–c** were obtained by protection of dialkynes **117a–c** with (3-cyanopropyl)dimethylsilyl chloride (CPDMSCl) (**118**). This protecting group was chosen to facilitate the separation of the mono- and diprotected products generated in this reaction. The two building blocks **116** and **119a–c** were subsequently assembled via the Sonogashira reaction producing differently substituted diynes **120a–c**. Deprotection of silyl groups in **120a–c** using TBAF furnished the key intermediates **121a–c** in moderate to good yields. Treatment of **121a–c** with Pd(PPh_3_)_4_ and copper iodide in THF in the presence of diisopropylamine gave the desired macrocycles **122a–c** (Scheme 17).

**Scheme 18:**
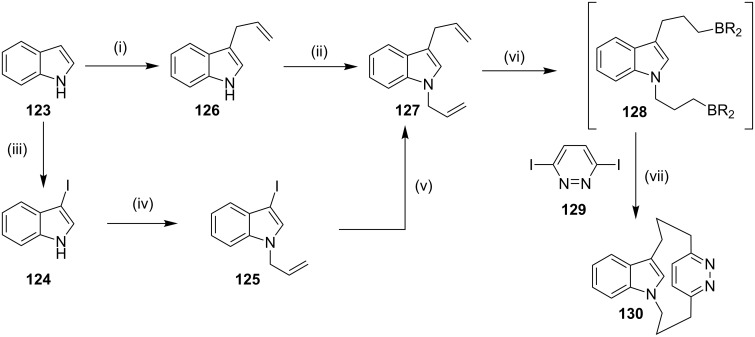
Synthesis of cyclophane **130** via Suzuki–Miyaura reaction as a key step. Reagents and conditions: (i) MeMgBr, allyl bromide, ether, 20 °C, overnight, 66%; (ii) KOH, allyl bromide, TBAB, rt, 6 h, 69%; (iii) KOH, I_2_, DMF, 20 °C, 0.45 h, 100%; (iv) KOH, allyl bromide, rt, 6 h, 98%; (v) *n*-BuLi, allyl bromide, 77%; (vi) 9-BBN, THF; (vii) **129**, Pd(PPh_3_)_2_Cl_2_, PPh_3_, Cs_2_CO_3_, dioxane, 65 °C, 5 h, 30%.

**Suzuki–Miyaura coupling:** Bodwell and Li [127] have reported the synthesis of the cyclophane **130** involving hydroboration and the Suzuki–Miyaura (SM) coupling [128–135] as key steps. 1,3-Diallylindole (**127**) was first synthesized in two steps from indole (**123**) by successive allylation at the 3 position to give **126** (66%) and later, *N*-allylation was carried out to afford the diallylindole **127** (69%, Scheme 18). A three-step (**123**→**124**→**125**→**127**) sequence was found to give a higher yield of the 1,3-diallylindole (**127**). Iodination of **123** gave the 3-iodoindole (**124**) quantitatively, which on *N*-allylation afforded **125** (98%). The treatment of compound **125** with *n*-BuLi followed by alkylation with allyl bromide gave diallylindole **127** (77%), which on further treatment with 9-BBN (6 equiv) gave the doubly hydroborated species **128**. Then, it was directly subjected to the Suzuki–Miyaura coupling reaction with 3,6-diiodopyridazine (**129**) and the desired cyclophane **130** was obtained (30%) as an oil (Scheme 18).

**Scheme 19:**
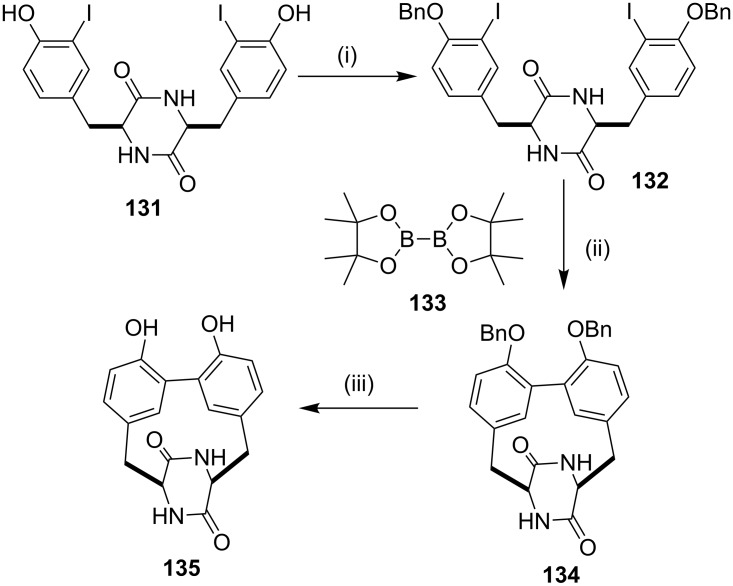
Synthesis of the mycocyclosin via Suzuki–Miyaura cross coupling. Reagents and conditions: (i) benzyl bromide (1.7 mmol), K_2_CO_3_ (1.7 mmol), DMF, 16 h, 76%; (ii) Pd(dppf)Cl_2_, **133** (1 equiv), DMSO, K_2_CO_3_, 90 °C for 16 h, 42%; (iii) pentamethylbenzene (1.1 mmol), TFA, 1 h, 74%.

In 2012, Hutton and co-workers [136] have synthesized a highly strained bicyclic framework of mycocyclosin (**135**) by utilizing the intramolecular Suzuki–Miyaura [137] cross-coupling reaction as a key step. The L,L-cyclodi(iodotryrosin) (**131**) was subjected to a benzylation reaction to give the protected compound **132** (76%). A one-pot Pd-catalyzed borylation and Suzuki–Miyaura coupling was employed to generate the cross-coupling product **134** (42%). Finally, deprotection of **134** was carried out with trifluoroacetic acid (TFA) in the presence of pentamethylbenzene to generate mycocyclosin (**135**, 74%) (Scheme 19).

**Scheme 20:**
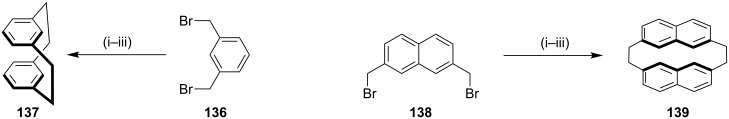
Synthesis of cyclophanes via Wurtz coupling reaction Reagents and conditions: (i) PhLi, Et_2_O, C_6_H_6_, reflux, 39%; (ii) Na, NaI (cat), PhBr (cat), Et_2_O, 12%; (iii) PhLi, Et_2_O, C_6_H_6_, 60 °C, 30 min, 20%.

**Wurtz coupling:** The Wurtz reaction is one of the oldest methods to form a C–C bond in organic synthesis. Baker and co-workers [138] have reported the synthesis of cyclophanes **137** and **139** by using the Wurtz coupling as a key step (Scheme 20).

#### Metathesis

**Scheme 21:**
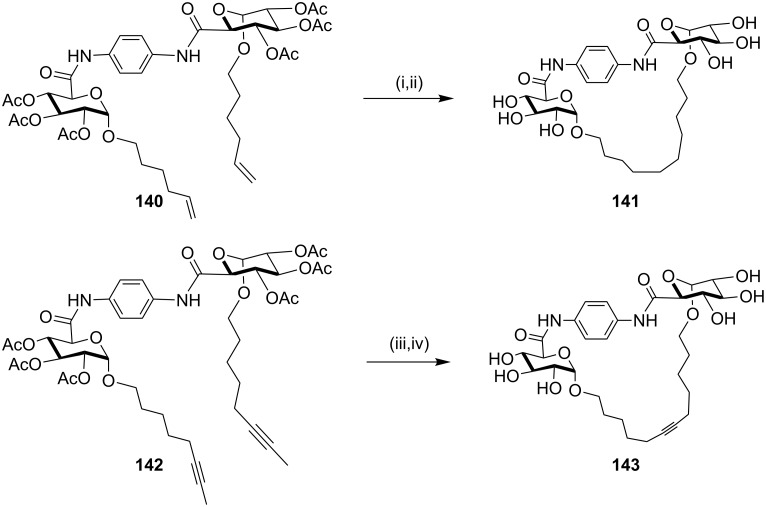
Synthesis of non-natural glycophanes using alkyne metathesis. Reagents and conditions: (i) G-I (**12**), CH_2_Cl_2_, 8 h; (ii) Pd/C (5 mol %), NaOMe/MeOH, 48%; (iii) Mo(CO)_6_, 2-fluorophenol, chlorobenzene, ∆; (iv) NaOMe/MeOH, 27%.

**Alkyne metathesis reaction:** In 2010, Murphy and Jarikote [139] have developed a useful protocol for assembling non-natural macrocyclic compounds containing carbohydrates. Compound **140** was prepared in several steps and was further subjected to the RCM with G-I (**12**) as a catalyst in CH_2_Cl_2_. Later, catalytic hydrogenation followed by deacetylation gave compound **141** (48%). Similarly, alkyne metathesis of compound **142** was carried out in the presence of Mo(CO)_6_ and 2-fluorophenol in chlorobenzene and heated under reflux to yield the cyclized product. The cleavage of the acetate groups with sodium methoxide in methanol gave the glycophane (a glycophane is a hybrid of carbohydrate and cyclophane) **143** (27%, Scheme 21).

**Figure 9:**
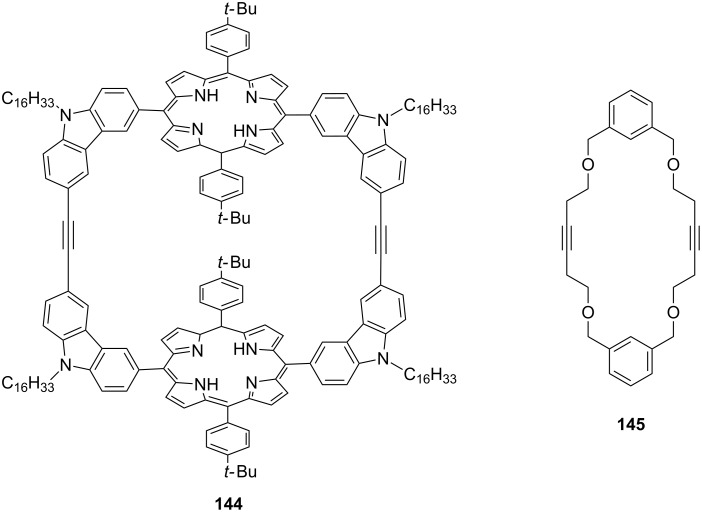
Synthesis of cyclophanes via ring-closing alkyne metathesis.

The synthesis of fullerene-related molecules with high binding affinity and/or high selectivity is an active research area due to the cost and energy demanding purification process and the poor processibility of the fullerenes. To this end, Zhang and co-workers [140] reported the synthesis of the bisporphyrin macrocycle **144** with an adaptable cavity by using alkyne metathesis with high efficiency. Tamm and co-workers [141] reported the synthesis of meta-cyclophane **145** at room temperature by ring-closing alkyne metathesis of 1,3-bis(3-pentynyloxymethyl)benzenes (Figure 9). This strategy has also been extended to ortho and para-derivatives.

**Scheme 22:**
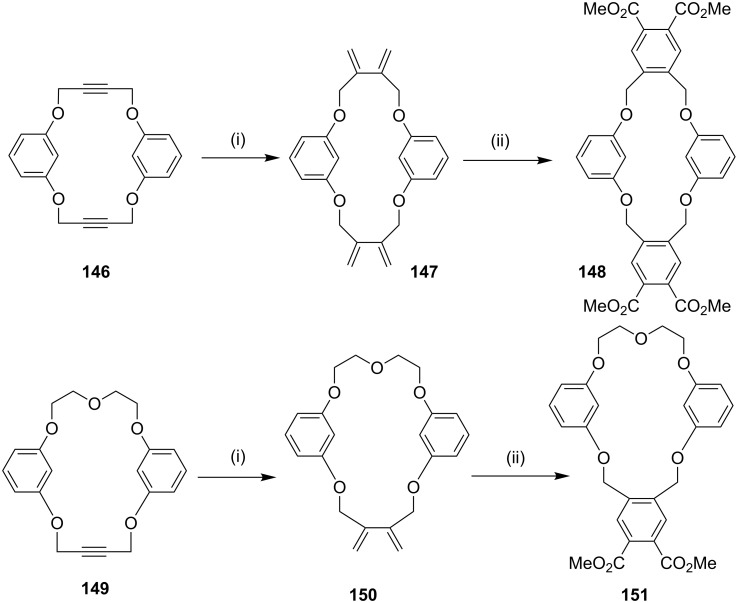
Synthesis of crownophanes by cross-enyne metathesis. Reagents and conditions: (i) G-II (**13**), 5 mol %, CH_2_Cl_2_, 24 h, rt, (**147,** 78%), (**150**, 82%); (ii) DMAD, PhMe, reflux, 24 h, DDQ, reflux, 30 h, (**148**, 78%), (**151**, 83%).

**Cross-enyne metathesis:** Recently, Kotha and Waghule [142] have synthesized diverse crownophanes by using a cross-enyne metathesis and Diels–Alder (DA) reaction as key steps. Here, the macrocycles **146** and **149** were subjected to a cross-enyne metathesis protocol with ethylene to generate the dienes **147** and **150**, respectively. These dienes were subjected to a DA reaction with different dienophiles followed by aromatization which gave the crownophanes (e.g., **148** and **151**) (Scheme 22).

**Scheme 23:**
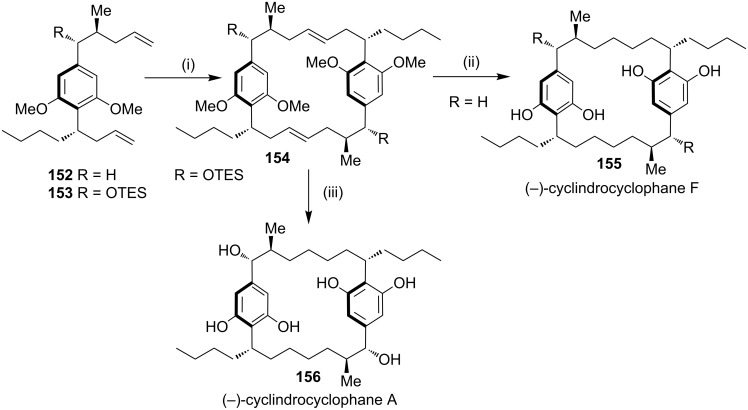
Synthesis of (−)-cylindrocyclophanes A (**156**) and (−)-cylindrocyclophanes F (**155**). Reagents and conditions*:* (i) G-I/G-II/Schrock catalyst, 50–80%; (ii) (a) H_2_, Pd/C; (b) BBr_3_ (84% over 2 steps); (iii) (a) TBAF, THF; (b) H_2_, PtO_2_; (c) PhSH, K_2_CO_3_, NMP (60% over 3 steps).

**Cross metathesis:** In 1992, (−)-cylindrocyclophane A (**156**) and (−)-cylindrocyclophane F (**155**) were isolated by Moore and co-workers [143] from a blue-green algae belonging to *Cylindrospermum licheniforme*. These paracyclophane derivatives exhibit potent cytotoxicity against the KB and LoVo tumor cell lines (IC_50_ = 2–10 μg/mL). On another occasion, Smith and co-workers have reported the synthesis of (−)-cylindrocyclophane A (**156**) and (−)-cylindrocyclophane F (**155**) [144]. The dialkenyl derivative **152** was subjected to dimerization involving cross-metathesis with G-I/G-II/Schrock catalysts which generated the cyclized product **154**. Subsequently, hydrogenation of the cyclophane **154** followed by minor functional group modification gave the natural products **155** and **156** (Scheme 23). Furthermore, the same group has reported the syntheses of (−)-cylindrocyclophanes A and F (**156**, **155**) by a RCM approach using different strategies.

**Scheme 24:**
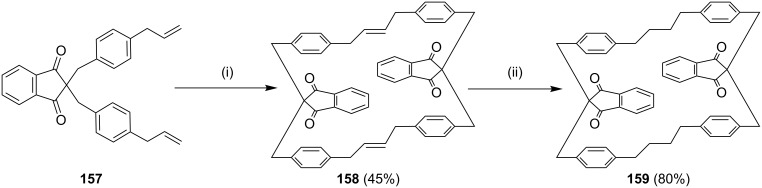
Synthesis of cyclophane **159** derivatives via SM cross-coupling and RCM. Reagents and conditions: (i) G-II (**13**), CH_2_Cl_2_ (0.002 M), 50 °C; (ii) H_2_, 10% Pd/C, CH_2_Cl_2_/MeOH, rt.

Kotha and co-workers [145] have synthesized cyclophanes by using 1,3-indanedione using freshly prepared KF-Celite followed by SM cross-coupling reaction with an excess amount of allylboronic acid pinacol ester and afforded the required diallyl derivative **157** in good yield. Surprisingly, when the dialkyl compound **157** was subjected to RCM, instead of the monomer, the dimeric cyclophane **158** was obtained which was further subjected to hydrogenation to deliver the saturated cyclophane derivative **159** (Scheme 24).

**Scheme 25:**
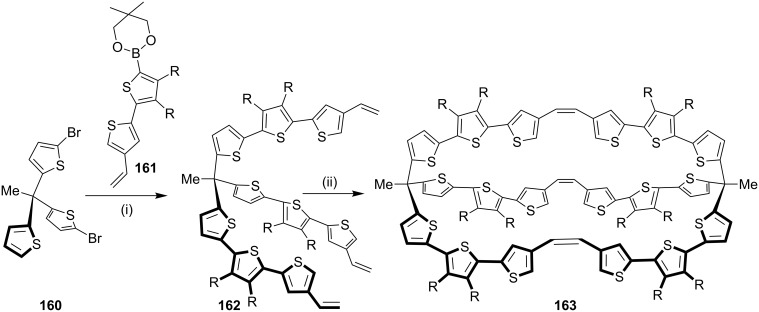
Sexithiophene synthesis via cross metathesis. Reagents and conditions: (i) **161**, Pd(PPh_3_)_4_, K_2_CO_3_, THF/PhMe/H_2_O; (ii) G-II (**13**), CH_2_Cl_2_, 27%.

To prepare π-conjugated three-dimensional molecules with potential isoelectronic properties and facile processibility, Kurata and co-workers [146] reported sexithiophene **163**, a bridged cage shaped compound (Scheme 25). Its synthesis involves a Suzuki–Miyaura coupling reaction followed by cross metathesis. The molecule shows a hypsochromic shift which indicates rigidity in the molecule compared with the other linear molecules.

**Scheme 26:**
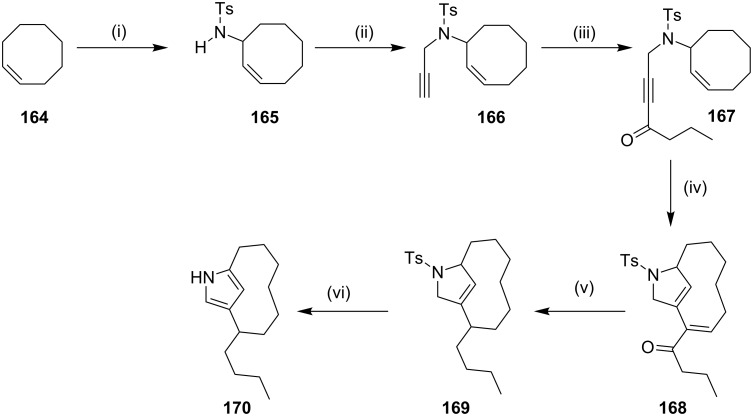
Synthesis of pyrrole-based cyclophane using enyne metathesis. Reagents and conditions: (i) Se, chloramine-T, 75%; (ii) NaH, THF, propargyl bromide, 92%; (iii) *n*-BuLi, −78 °C, ZnCl_2_, −30 °C, butanoyl chloride, rt, 82%; (iv) PtCl_2_ (5 mol %), 66 h, 20 °C, 79%; (v) (a) Bu_3_SnH, Pd(0), HBF_4_, 94%; (b) LiAlH_4_, 96%; (c) PhOC(S)Cl, 95%; (d) Bu_3_SnH, AlBN, 64%; (vi) (CH_2_)_3_(NH_2_)_2_, KH, 3 h, KAPA, 75%.

**Enyne metathesis:** In 1998, Fürstner and co-workers [147] have employed platinum(II)-catalyzed enyne metathesis as a key step to form cyclophane ring systems which are found in streptorubin B and metacycloprodigiosin [148–150]. In this context, the cyclooctene **164** was reacted with the intermediate formed in situ from chloramine-T and elemental selenium [151] and yielded the allylic amine derivative **165** (75%). An N-alkylation with propargyl bromide gave the enyne product **166** (92%), which on further acylation of terminal alkyne with butanoyl chloride delivered compound **167** (82%). Then, it was subjected to an enyne metathesis with simple platinum salts such as PtCl_2_ and PtCl_4_ to give product **168** (79%). A subsequent reduction of the α,β-unsaturated ketone delivered the compound **169** (64%). Finally, aromatization of compound **169** by using potassium 3-aminopropylamide (KAPA) gave compound **170** (75%) (Scheme 26).

**Scheme 27:**
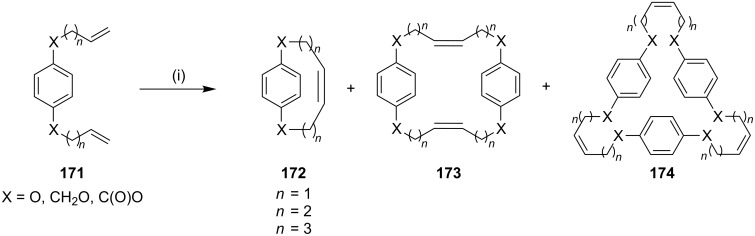
Synthesis of macrocyclic derivatives by RCM. Reagents and conditions: (i) G-I/G-II, CH_2_Cl_2_, 0.005 M, 45 °C, 14–22 h, 60–76%.

**Ring-closing metathesis (RCM):** In 2003, Tae and Yang [152] have reported an efficient macrocyclization of various alkenyl derivatives **171** via RCM/CM using G-I (**12**) or G-II (**13**) under high dilution conditions to obtain the [*n*], [*n*,*n*] and [*n*,*n*,*n*]paracyclophanes **172–174**. Compounds with a short alkenyl chain gave mainly [*n*,*n*] and [*n*,*n*,*n*]paracyclophanes (**173** and **174**) by a dimerization or trimerization sequence. When the compound has a alkenyl chain of sufficient length the [*n*]paracyclophane **172** was obtained by an intramolecular cyclization (Scheme 27).

**Scheme 28:**
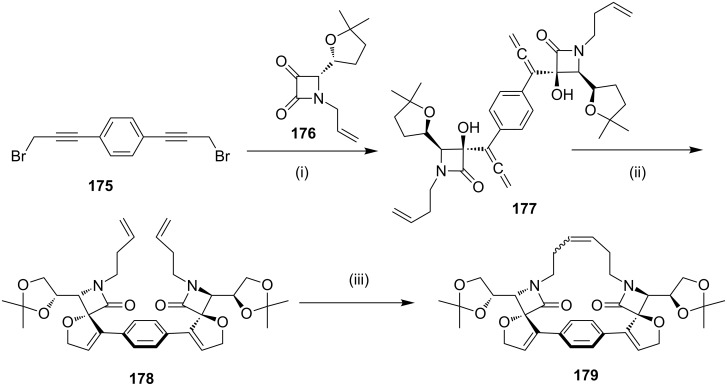
Synthesis of enantiopure β-lactam-based dienyl bis(dihydrofuran) **179**. Reagents and conditions: (i) indium, THF/saturated aq NH_4_Cl 1:5, 24 h; (ii) 5 mol % AuCl_3_, CH_2_Cl_2_, rt, 3 h; (iii) 10 mol % Ru(II) or Ru(III) catalyst, CH_2_Cl_2_ (high dilution conditions).

Alcaide and co-workers [153] have reported the synthesis of different bis(dihydrofuryl)cyclophane scaffolds **179** from carbonyl compounds. 1,4-Bis(3-bromoprop-1-ynyl)benzene (**175**) was reacted with azetidine-2,3-diones **176** under eco-friendly reaction conditions to generate bis(allene) **177**. Compound **177** was then converted into bis(dihydrofuran) **178** by using AuCl_3_. Macrocyclization of **178** was carried out by using a Ru(II) or Ru(III) catalyst to generate **179** as a mixture of *E*/*Z* isomers (Scheme 28).

**Scheme 29:**
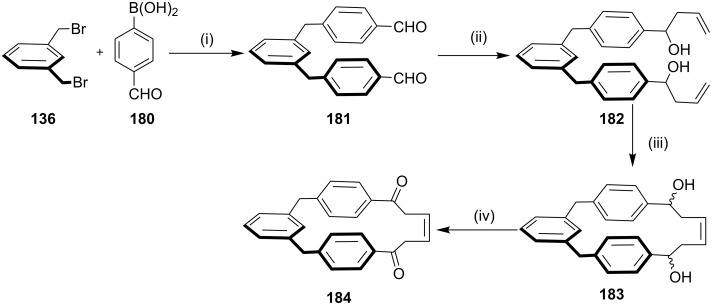
Synthesis of a [1.1.6]metaparacyclophane derivative **183** via SM cross coupling. Reagents and conditions: (i) Pd(PPh_3_)_4_, Na_2_CO_3_, THF/water, reflux, 12 h, 80%; (ii) indium, allyl bromide, DMF; (iii) G-I (**12**), CH_2_Cl_2_; (iv) PCC, CH_2_Cl_2_.

In the literature, there are limited reports on the preparation of cyclophane derivatives by a combination of the Suzuki–Miyaura (SM) coupling and an RCM as key steps. Kotha and Mandal [135] reported a new approach to assemble [1.1.6]metaparacyclophane derivative **183** via the SM cross coupling and an RCM as key steps. In this regard, the α*,*α'-dibromo-*m*-xylene (**136**) was treated with arylboronic acid **180**, to give the dialdehyde **181** which on reaction with indium-mediated Grignard addition reaction gave diolefin **182**. Later RCM of diolefin **182** delivered cyclophane **183**. Subsequent oxidation of diol **183** gave [1.1.6]metaparacyclophane derivative **184** (Scheme 29).

**Scheme 30:**
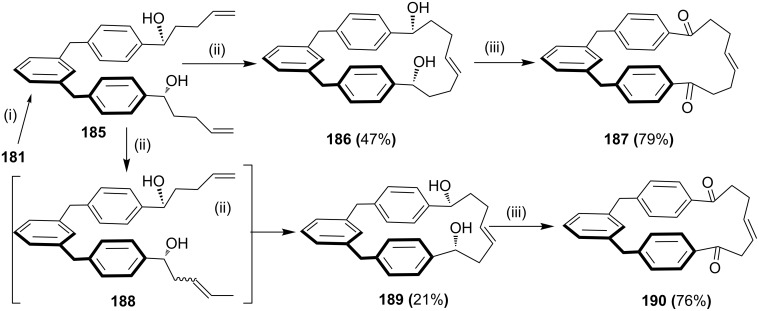
Synthesis of a [1.1.6]metaparacyclophane derivative **190** via SM cross coupling. Reagents and conditions: (i) Mg, Et_2_O, 4-bromobut-1-ene; (ii) G-II (**13**, 10 mol %), CH_2_Cl_2_; (iii) PCC, CH_2_Cl_2_, rt.

Using the same approach, a butenyl Grignard reagent was added to compound **181** to generate diol **185**. Surprisingly, after the addition of G-II catalyst **13**, the two RCM products **186** and **189** were obtained [135]. The outcome of product **189** was explained on the basis of a tandem isomerization of a terminal double bond followed by the macrocyclization with G-II (**13**). Finally, the oxidation of diols **186** and **189** generated cyclophanes **187** and **190**, respectively (Scheme 30).

**Scheme 31:**
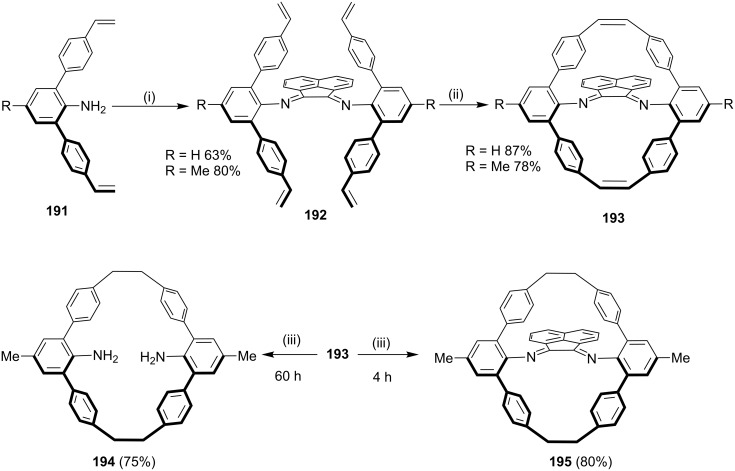
Template-promoted synthesis of cyclophanes involving RCM. Reagents and conditions: (i) acenaphthenequinone, *p*-TSA, C_6_H_6_; (ii) G-II (**13**), CH_2_Cl_2_ (0.002 M), 50 °C; (iii) Pd/C (10 mol %), H_2_, CH_2_Cl_2_/MeOH, rt.

Guan and coworkers [154] have reported a novel synthetic approach to cyclophanes by using a template-promoted cyclization involving the RCM as a key step. This approach proceeded via the condensation of compound **191** with acenaphthenequinone in the presence of *p*-TSA to deliver the RCM precursor **192**, which facilitate the cyclization protocol with G-II (**13**) as a catalyst to generate cyclophane derivative **193** containing an α-diimine functionality. Subsequently, the hydrogenation of **193** gave cyclophane **195**. The removal of the template under hydrogenolytic conditions gave the macrocyclic compound **194** (Scheme 31).

**Scheme 32:**
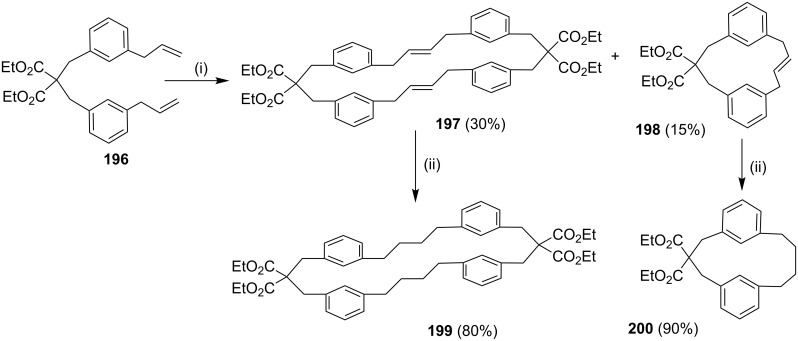
Synthesis of [3.4]cyclophane derivatives **200** via SM cross coupling and RCM. Reagents and conditions: (i) G-II (**13**), CH_2_Cl_2_ (0.002 M), 50 °C; (ii) H_2_, 10% Pd/C, CH_2_Cl_2_/MeOH, rt.

In continuation of earlier work [145], Kotha and co-workers have demonstrated an interesting strategy to assemble [3.4]cyclophane derivative **197** by using the SM cross coupling and an RCM as key steps. The commercially available active methylene compound diethyl malonate was alkylated with a benzyl bromide derivative followed by the SM cross coupling to give dialkyl **196**. Subsequently, an olefin metathesis with G-II (**13**) as a catalyst delivered dimeric **197** and monomeric **198** cyclophane derivatives. Later, the hydrogenation of **197** and **198** gave the corresponding saturated [3.4]cyclophane derivatives **199** and **200**, respectively (Scheme 32).

**Figure 10:**
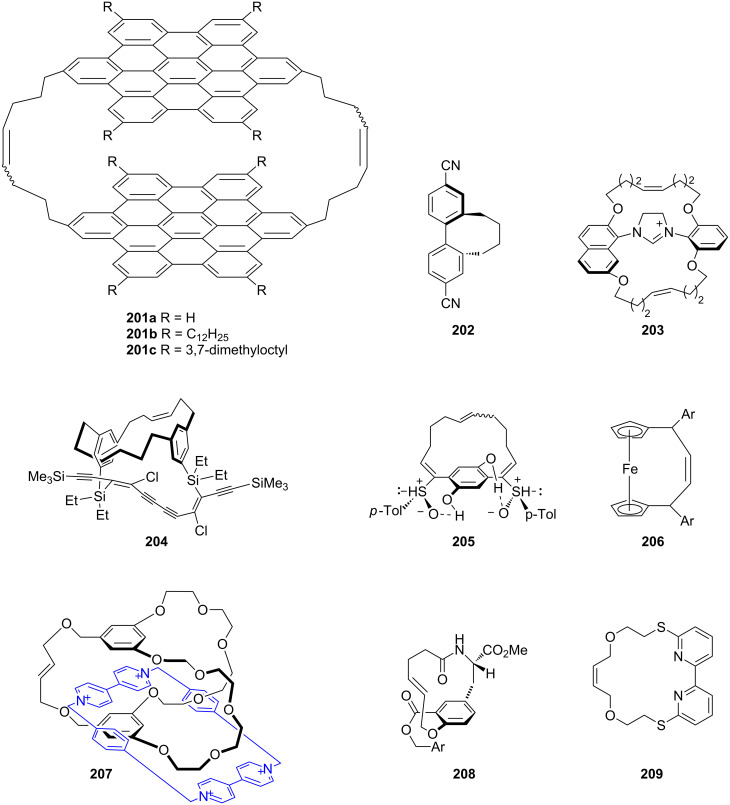
Examples for cyclophanes synthesized by RCM.

Müllen and co-workers [155] have synthesized hexa-peri-hexabenzocoronene cyclophane **201a–c**. They studied their properties by carrying out differential scanning calorimetry (DSC), optical microscopy, wide-angle X-ray scattering (WAXD), and scanning tunneling microscopy (STM). Tunneling spectroscopy reveals a diode-like behavior which introduces a high caliber of these molecular complexes. The RCM protocol has been successfully employed to generate a series of dicyanobiphenylcyclophanes **202** which are useful as *n*-type semiconductors [156]. Winkelmann and co-workers [157] have synthesized chiral concave imidazolinium salts **203** as precursors to chiral concave N-heterocyclic carbenes. Molecular encapsulation was achieved by using double RCM to generate insulated oligoynes **204**. Here, the masked hexayne plays an important role to lock the flanking chains [158]. The synthesis of planer chiral cyclophanes is a difficult task owing to the flipping of the ansa-chain present in these molecules. Suzuki and co-workers [159] have reported the synthesis of enantiomerically pure planar-chiral [10]- and [12]paracyclophanes **205**, which will serve as useful intermediates for the synthesis of various other cyclophane derivatives. Literature reports demonstrate the extensive use of RCM in the synthesis of different metallophanes involving ferrocenophane (e.g., **206**) [160] and other metallophanes [161–164]. The synthesis of mechanically interlocked molecules such as catenanes and rotaxanes which are used to assemble molecular machines, sensors and nanomaterials is a challenging task. Huang and co-workers [165] have reported a taco complex template method to synthesize a cryptand/paraquat [2]rotaxane and [2]catenane (e.g., **207**) by using RCM as a key step. Structural features and interesting bioactivity of the hirsutellones have grabbed the attention of synthetic chemists. Liu and co-workers [166] have constructed the [10]paracyclophane **208** (skeleton of hirsutellones) via RCM. The 2,2’-bipyridine unit is an interesting building block due to its use in chelating ligands, as a binding agent and also a useful template in supramolecular chemistry, Rykowski and co-workers [167] have synthesized azathiamacrocycle **209** using RCM (Figure 10).

**Scheme 33:**
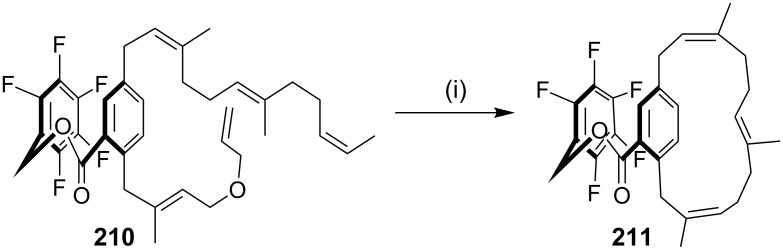
Synthesis of the longithorone C framework assisted by fluorinated auxiliaries. Reagents and conditions: (i) Blechert catalyst (**17**, 10 mol %), Ti(iOPr)_4_, CH_2_Cl_2_, 4 h, 39%.

Collins and co-workers [168] have reported the application of auxiliaries that engage in quadrupolar interactions in a total synthesis of a macrocyclic portion of longithorone C. To investigate the macrocyclization with the pentafluorobenzyl ester auxiliary, ester **210** was synthesized in a multistep process and then subjected to olefin metathesis to deliver the macrocycle using the Blechert catalyst **17**. The treatment of the pentafluorophenyl benzyl ester **210** with catalyst **17** in toluene afforded the rigid macrocycle **211** (39%, Scheme 33).

**Scheme 34:**
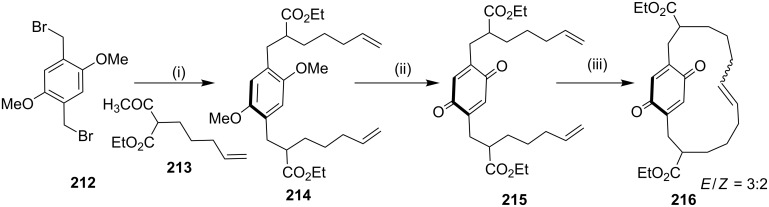
Synthesis of the longithorone framework via RCM. Reagents and conditions: (i) **213**, NaH, THF, rt, 10–15 h; (ii) CAN/SiO_2_, H_2_O, CH_2_Cl_2_, 5 min, rt, 67%; (iii) **13**, (5 mol %), PhMe, reflux, 10 h, 71%.

Kotha and Shirbhate [169] have reported the longithorone framework by using RCM as a key step. Dibromo compound **212** was reacted with monoalkylated ethyl acetoacetate **213** in the presence of NaH to deliver bis-alkyated product **214**, followed by an oxidation the quinone derivative **215** (67%) was obtained. Next, the quinone **215** was subjected to RCM to generate the cyclized product **216** (71%, Scheme 34).

**Scheme 35:**
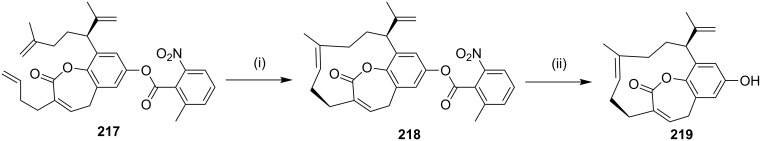
Synthesis of floresolide B via RCM as a key step. Reagents and conditions: (i) G-II (13, 0.1 equiv), 0.5 mM in CH_2_Cl_2_, 40 °C, 15 min; (ii) K_2_CO_3_ (10.0 equiv), MeOH/H_2_O 1:1, 25 °C, 2 h, 90%.

Nicolaou and Xu [170] assembled the floresolide B **219** via RCM as a key step. Compound **217** underwent cyclization in the presence of G-II (**13**) in DCM heated under reflux to generate the two isomers of **218** (89%). Subsequently, the cleavage of the nitrobenzoate group with K_2_CO_3_ in MeOH gave the floresolide B **219** (Scheme 35).

**Scheme 36:**
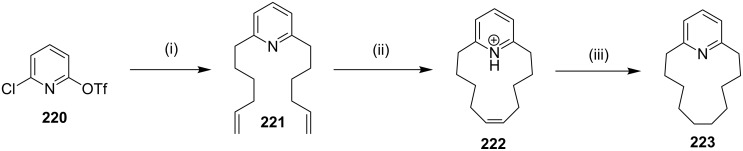
Synthesis of normuscopyridine (**223**) by the RCM strategy. Reagents and condition: (i) Mg, THF, hexenylmagnesium bromide, Fe complex **18** (10 mol %), THF/NMP, 0 °C, 75%; (ii) (a) HCl, Et_2_O; (b) Ru catalyst **17** (10 mol %), CH_2_Cl_2_, reflux, 14 h; (iii) (a) H_2_ (50 atm.), 70 °C; (b) aq sat. NaHCO_3_, 68%.

Fürstner and Leitner [171] have reported the synthesis of the normuscopyridine (**223**) by a cross-coupling reaction and an RCM as key steps. The treatment of the pyridine derivative **220** with an excess amount of the 5-hexenylmagnesium bromide in the presence of a catalytic amount of iron complex **18** as the precatalyst provides the dialkylation product **221** (75%). The treatment of the hydrochloride solution of **221** with Ru catalyst **17** in a dilute CH_2_Cl_2_ solution gave the cycloalkene **222** which on subsequent hydrogenation yielded the targeted normuscopyridine (**223**, 68%, Scheme 36).

**Scheme 37:**
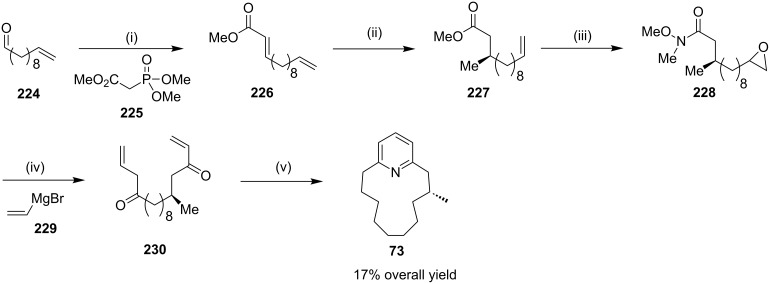
Synthesis of muscopyridine (**73**) via RCM. Reagents and conditions: (i) **225**, NaH, THF, 0 °C to rt, 1.5 h, 95%; (ii) CuI (5 mol %) (*R*)-tol-BINAP (7.5 mol %), *t*-Bu_2_O, MeMgBr, −20 °C, 1 h, rt, 15 h, 77%; (iii) (a) iPrMgCl, THF, −10 °C to rt, 20 min, 89%; (b) NHMeOMe, CH_2_Cl_2_, *m*-CPBA, rt, 19 h, 96%; (iv) **229**, cat. CuI, DMP, −10 °C, 1 h, 68%; (v) (a) G-H-II (10 mol %), CH_2_Cl_2_, 55 °C, (b) NH_4_OAc, AcOH, EtOH, 96 h, 42%.

Donohoe and coworkers [172] have reported the synthesis of muscopyridine (**73**) by RCM as a key step. The Wadsworth–Emmons olefination of the commercially available undecenal **224** provided acrylate **226**, which was subjected to enantioselective copper-catalyzed conjugate addition with a methyl Grignard reagent involving (*R*)-tol-BINAP ligand to generate ester **227** in good yield and high enantiopurity. This intermediate was then converted to the key metathesis precursor involving a three step sequence of a Weinreb amide formation **228**, epoxidation, and double addition of the vinyl Grignard **229** to generate the advanced intermediate **230**. Finally, RCM of diolefin **230** under high dilution conditions afforded muscopyridine (**73**) (Scheme 37).

**Scheme 38:**
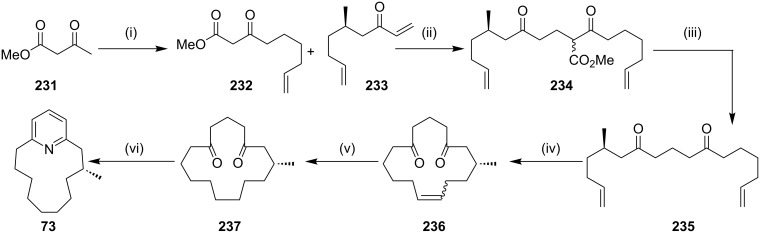
Synthesis of muscopyridine (**73**) via RCM strategy. Reagents and conditions: (i) NaH, *n*-BuLi, 5-bromo-1-pentene, rt, 2.5 h, 60%; (ii) **233**, K_2_CO_3_, (*n*-Bu)_4_NI, rt, 1 h, 93%; (iii) LiCl, DMPU, 120 °C, 7 h, 72%; (iv) G-I (**12**), CH_2_Cl_2_, 40 °C, 16.5 h, 90%; (v) Pd/C, H_2_, EtOH, rt, 5 h, 98%; (vi) NH_2_OH·HCl, 150–160 °C, 16 h, 61%.

Hagiwara and co-workers [173] have synthesized muscopyridine starting with methyl acetoacetate (**231**). They treated **231** with 5-bromo-1-pentene to generate keto ester **232** (60%). The coupling of keto ester **232** with vinyl ketone **233** under phase-transfer catalysis conditions generated the new keto ester **234** (93%), which on treatment with lithium chloride at 120 °C in dimethyl propylene urea (DMPU) gave dione **235** (72%). An RCM sequence of compound **235** in the presence of G-I (**12**) catalyst gave the RCM product **236**. A subsequent catalytic hydrogenation generated the saturated dione **237**. Finally, the pyridine ring has been introduced by reacting dione **237** with hydroxylamine hydrochloride in a sealed tube to furnish muscopyridine (**73**, 61%, Scheme 38).

**Scheme 39:**
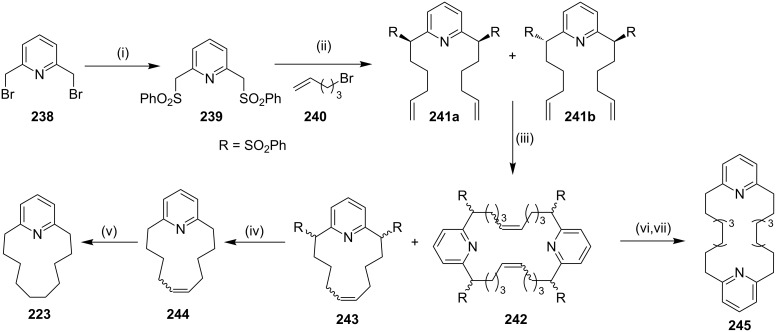
Synthesis of pyridinophane derivatives **223** and **245**. Reagents and conditions: (i) PhSO_2_Na, TBAB, CH_3_CN, reflux, 12 h, 87%; (ii) **240**, NaH, THF, rt, 24 h, 65%; (iii) G-I (**12**) (5 mol %), CH_2_Cl_2_, rt, 48 h, **243** (51%), **242** (20%); (iv) Mg/TMSCl, 1,2-dibromoethane, EtOH, 12 h, 80%; (v) H_2_, EtOAc, Pd/C, rt, 12 h, 84%; (vi) Mg/TMSCl, 1,2-dibromoethane, EtOH, 12 h; (vii) H_2_, EtOAc, Pd/C, rt, 12 h, (two steps 64%).

Normuscopyridine has been also obtained by an RCM approach. To this end, commercially available 2,6-lutidine dibromide **238** was reacted with sodium benzenesulfinate to deliver 2,6-bis(benzenesulfonylmethyl)pyridine (**239**) in quantitative yield. Next, bis-sulfone **239** was reacted with 5-bromo-1-pentene (**240**) in the presence of NaH to give an inseparable mixture of *cis* and *trans*-sulfones **241a** and **241b**, respectively. An RCM sequence of these sulfones in the presence of the G-I (**12**) catalyst gave cyclophane **243** (51%) and dimeric cyclophane **242** (20%, Scheme 39) [174]. The reduction of the sulfonyl group with Mg/ethanol in the presence of 1,2-dibromoethane aided by TMSCl afforded cyclophane derivative **244** (80%). Subsequently, the hydrogenation of the double bond with Pd/C under a H_2_ atmosphere gave normuscopyridine (**223**, 84%). Similar reaction conditions were employed with the dimeric product **242**, to generate the macrocyclic pyridinophane **245** (64%).

**Scheme 40:**
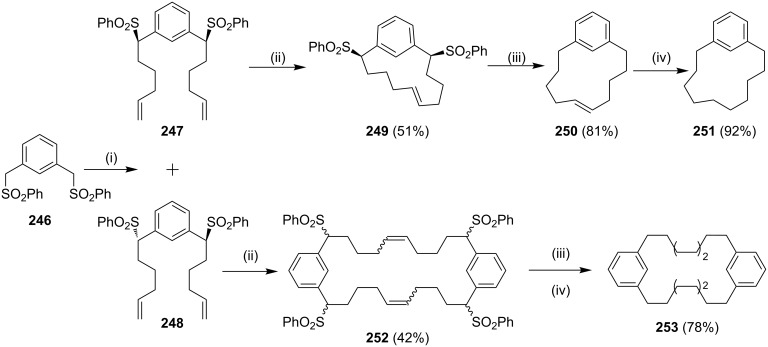
Synthesis of metacyclophane derivatives **251** and **253**. Reagents and conditions: (i) **240**, NaH, THF, rt, 24 h; (ii) G-I (**12**, 5 mol %), CH_2_Cl_2_, rt, 48 h, **247** (29%), **248** (30%); (iii) Mg/TMSCl, 1,2-dibromoethane, EtOH, 12 h; (iv) H_2_, EtOAc, Pd/C, rt, 12 h.

It is interesting to note that when the same strategy was applied with a benzene analogue, dipentenylation of bis-sulfone **246** gave compounds **247** and **248**, which were easily separable by column chromatography [174]. Moreover, it was observed that *cis*-sulfone generates the monomeric cyclophane **249** during the metathesis as confirmed by single crystal X-ray diffraction data while the *trans*-sulfone gave the dimer **252**. Finally, the desulfonylation followed by the hydrogenation sequence of **249** and **252** generate the cyclophanes **251** and **253**, respectively (Scheme 40).

**Scheme 41:**
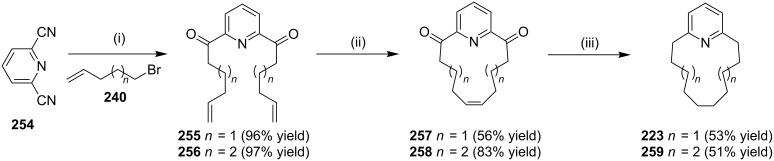
Synthesis of normuscopyridine and its higher analogues. Reagents and conditions: (i) alkenyl bromide, Mg, ether, H_2_O/H^+^; (ii) G-II (**13**, 5 mol %), PhMe, reflux; (iii) N_2_H_4_, K_2_CO_3_, ethylene glycol, 180 °C.

With regard to the synthesis of cyclophane, Kotha and co-workers [174] have demonstrated another synthetic route to normuscopyridine (**223**) involving a short synthetic sequence. This route involves the reaction of dicyanopyridine **254** with alkenylmagnesium bromide to generate **255** and **256**. Further, these compounds were cyclized with the aid of the G-II catalyst **13** to generate the corresponding RCM products **257** and **258** , respectively. The removal of the two carbonyl groups and the hydrogenation of the double bond was accomplished in a one-pot reaction under Wolff–Kishner reaction conditions to generate **223** and **259**, respectively (Scheme 41).

#### Cycloaddition reactions

**Scheme 42:**
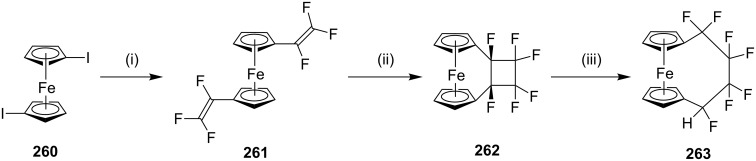
Synthesis of fluorinated ferrocenophane **263** via a [2 + 2] cycloaddition. Reagents and conditions: (i) Pd(OAc)_2_, PPh_3_, CF_2_CFZnCl, THF, 5 h, reflux, 95%; (ii) PhMe, 110 °C, 2 h , 5%; (iii) K_3_Fe(CN)_6_, KF, H_2_O, *t*-BuOH, rt, 1 h, 67%.

**[2 + 2] Cycloaddition:** Roemer and Lentz [175] have reported the synthesis of fluorinated ferrocenophanes from 1,10-bis(trifluorovinyl)ferrocene and 1,4-(1,10-ferrocenediyl)-1,1,2,2,3,3,4-heptafluorobutane. The authors have reported a [2 + 2] cycloaddition reaction under thermal conditions. 1,10-Bis(trifluorovinyl)ferrocene (**261**) was synthesized starting with diiodoferrocene **260** by Negishi-type coupling. Compound **261** was subjected to a [2 + 2] cycloaddition sequence to generate cyclobutane derivative **262**. Finally, the ring opening occurs with catalytic amounts of potassium hexacyanoferrate(III) in the presence of KF to deliver the fluorinated ferrocenophane **263** (Scheme 42).

**Scheme 43:**
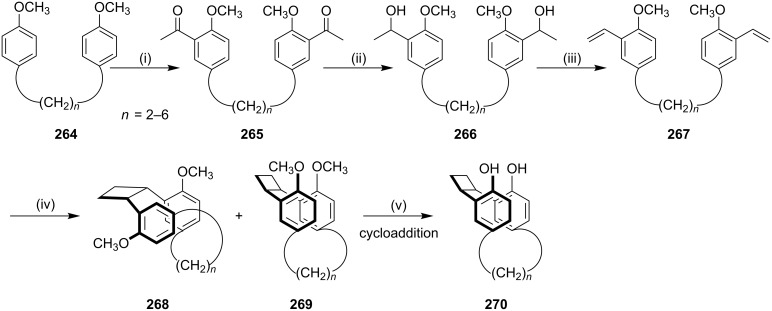
Synthesis of [2.*n*]metacyclophanes **270** via a [2 + 2] cycloaddition. Reagents and conditions: (i) Ac_2_O, AlCl_3_, PhNO_2_, Cl_2_CHCHCl_2_, rt, 12 h, 58–93%; (ii) LiAlH_4_, THF, rt, 1 h, ~100%; (iii) PyHOTs, C_6_H_6_, reflux, 5 d, 72–92%; (iv) *h*v, C_6_H_6_, rt, 26–92 h, 61–87%; (v) BBr_3_, CH_2_Cl_2_, rt, 12 h, 70–80%.

Okada and Nishimura [6] have reported the synthesis of *syn*-[2.*n*]metacyclophane **270** as a key building block for the synthesis of calix[4]arene. Here, α,ω-bis(*p*-methoxyphenyl)alkanes **264** were used as starting materials. Compound **264** was treated with acetic anhydride and AlCl_3_ in nitrobenzene and 1,1,2,2-tetrachloroethane to generate diketone **265** in 58–93% yield. Diketone **265** was then treated with LAH to generate diol **266** (72–92%). The dehydration of diol **266** with pyridinium *p*-toluenesulfonate in benzene gave diolefin **267**. [2 + 2] Photocycloaddition of diolefin **267** was carried out by irradiation with a 400 W high-pressure Hg lamp (Pyrex filter) in benzene for 26–92 h. After evaporation, **268** and [2.*n*]metacyclophane **269** were isolated (61–87%). Finally, demethylation of compound **269** with BBr_3_ in CH_2_Cl_2_ gave cyclophane **270** (Scheme 43).

**Scheme 44:**
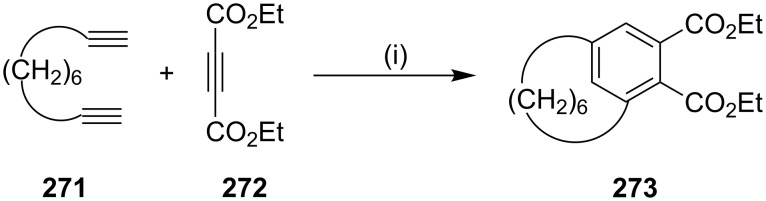
Synthesis of metacyclophane **273** by a [2 + 2 + 2] co-trimerization. Reagents and conditions: (i) [Rh(cod)_2_]BF_4_/H8-BINAP, CH_2_Cl_2_, 1 h, rt, 50%.

**[2** + **2** + **2] Co-trimerization:** In 2003, Tanaka and Shirasaka [176] have reported a one-step synthesis of [6]metacyclophane **273** by a [2 + 2 + 2] co-trimerization of two different alkynes with a high chemo- and regioselectivity. The Rh(I)/H8-BINAP complex catalyzed the partially intermolecular cyclotrimerization of 1,9-decadiyne (**271**) and diethyl acetylenedicarboxylate (**272**) to give [6]metacyclophane derivative **273** (Scheme 44) [177]. This approach is also applicable to synthesize various polyether-based cyclophanes. In this report, they have synthesized various polyether containing cyclophanes by a cross-cyclotrimerization catalyzed by a cationic rhodium(I)/H8-BINAP complex as a key step. The ether linked α,ω-diynes and dimethyl acetylenedicarboxylate were treated with the Ru catalyst to deliver the metacyclophane in a regioselective manner. The ratio of para, meta, and orthocyclophane formation depends on the chain length of the diynes employed (Scheme 44).

**Scheme 45:**
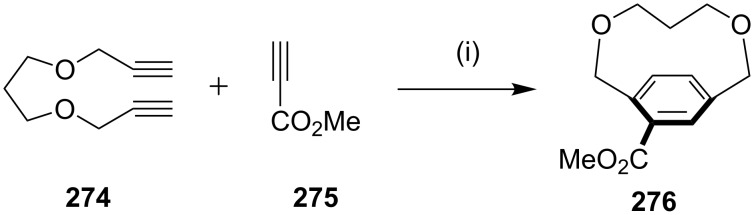
Synthesis of paracyclophane **276** via a [2 + 2 + 2] cycloaddition reaction. Reagents and conditions: (i) [Rh(cod)_2_]BF_4_/H8-BINAP, (5–10 mol %), CH_2_Cl_2_, rt, 1 h, (18% yield, 75% ee).

Tanaka and co-workers [178] demonstrated a useful approach to strained dioxa[7]paracyclophane **276** by the application of a [2 + 2 + 2] cycloaddition sequence (Scheme 45). To this end, [2 + 2 + 2] cycloaddition of 1,10-diyne **274** was carried out with methyl propiolate (**275**) in the presence of a cationic rhodium(I)-(*S*)-BINAP complex (10 mol %) as a catalyst. The desired [2 + 2 + 2] cycloaddition was carried out at room temperature to generate dioxa[7]paracyclophane **276** with a moderate *ee* value. The effect of biaryl bis(phosphine) ligands was examined, and it revealed the use of (*S*)-H8-BINAP afforded the cyclophane **276** with a good yield and optimum *ee* value.

**Scheme 46:**
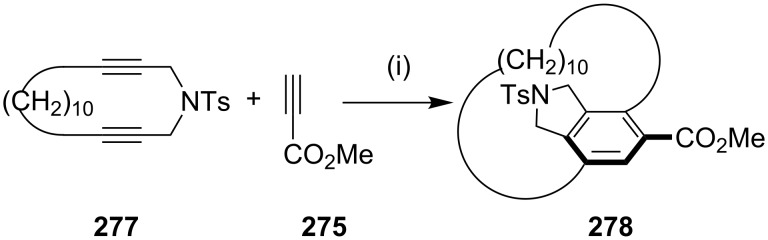
Synthesis of cyclophane **278** via a [2 + 2 + 2] cycloaddition reaction. Reagents and conditions: (i) 5–20 mol % [Rh(cod)_2_]BF_4_/(*S*,*S*)-bdpp, CH_2_Cl_2_, rt, 16 h, (91% ee).

Similarly, they also reported the synthesis of the planar-chiral carba-paracyclophane **278** by using the cationic rhodium(I)/(*S*,*S*)-bdpp-catalyzed [2 + 2 + 2] cycloaddition of cyclic diyne **277** with terminal methyl propiolate (**275**) under high substrate concentration conditions (Scheme 46) [179].

**Scheme 47:**
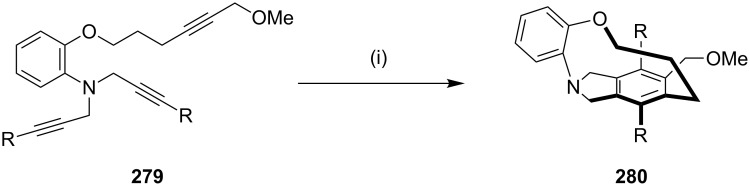
Synthesis of cyclophane **280** via a [2 + 2 + 2] cycloaddition. Reagents and conditions: (i) [(Rh(cod)(*S*,*S*)-Me-duphos)]OTf (10 mol %), DCE, 120 °C, (77% yield, 98 ee).

Shibata and co-workers [180] have synthesized chiral tripodal cage compounds (e.g., **280**) by using a [2 + 2 + 2] cycloaddition reaction of branched triynes (Scheme 47). The best results for a cycloaddition were observed when triyne **279** was added dropwise over a period of 10 min to a solution of a chiral catalyst at elevated temperature (120 °C). Also, highly enantioselective intramolecular reactions of different nitrogen-branched triynes were carried out to obtain diverse cyclophanes (Scheme 47).

**Scheme 48:**
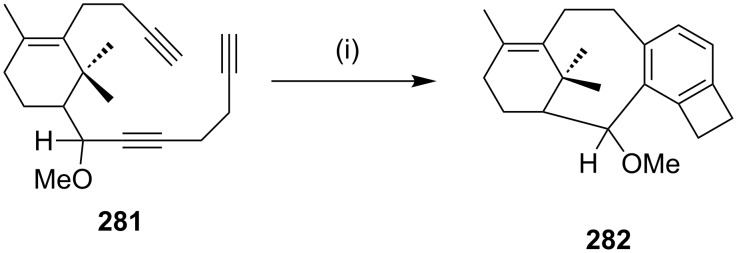
Synthesis of taxane framework by a [2 + 2 + 2] cycloaddition. Reagents and conditions: (i) Cp(CO)_2_ (5 mol %), xylene, *h*v, reflux.

Malacria and co-workers [181] have demonstrated an efficient use of a [2 + 2 + 2] cycloaddition reaction to generate the tetracyclic structure **282** related to taxane skeleton (Scheme 48).

**Scheme 49:**
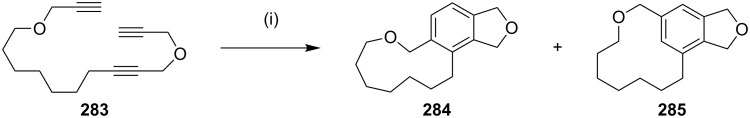
Synthesis of cyclophane **284** and **285** via a [2 + 2 + 2] cycloaddition reaction. Reagents and conditions: (i) RhCl(cod)_2_tppts (2.5 mol %), H_2_O/Et_2_O, 20 h.

Ohsima and co-workers [182] have reported a rhodium-catalyzed [2 + 2 + 2] cyclotrimerization of triynes **283** in a water-organic biphasic system. The biphasic system provides dilute reaction conditions suitable for macrocyclization. Selective cross-annulation between hydrophobic diynes and hydrophilic alkynes was achieved to generate ortho- and metacyclophane **284** and **285** (Scheme 49).

**Scheme 50:**
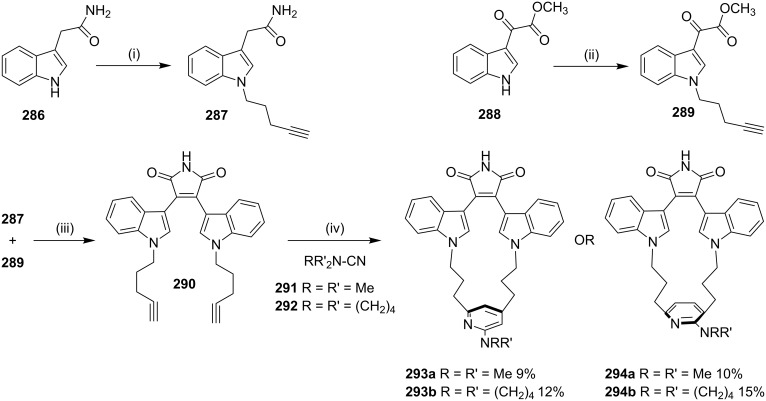
Synthesis of pyridinophanes **293a**,**b** and **294a**,**b** via a [2 + 2 + 2] cycloaddition. Reagents and conditions: (i) 5-chloro-1-pentyne, NaH, DMF, 0–55 °C, 12 h, 90%; (ii) 5-chloro-1-pentyne, Cs_2_CO_3_, DMF, 10 h, 59%; (iii) KO*t*-Bu, THF, 0–23 °C, 6 h, 63%; (iv) CpCo(CO)_2_, 1,4-dioxane, 105–110 °C, 24 h.

Maryanoff and co-workers [183] have synthesized the bis(indolyl)maleimido pyridinophanes via a [2 + 2 + 2] cycloaddition reaction as a key step. In this regard, indole-3-acetamide (**286**) was treated with 5-chloro-1-pentyne and NaH in DMF to deliver compound **287**. Then, indole-3-glyoxylate **288** was converted to N-alkylated derivative **289** by the treatment with 5-chloro-1-pentyne in the presence of cesium carbonate. The maleimide condensation of **287** and **289** was carried out in the presence of KO*t*-Bu at 0–23 °C to give the α,α'-diyne substrate **290** (63%, Scheme 50). Next, the diyne **290** was reacted with *N*,*N'*-dimethylcyanamide (**291**) or **292** and CpCo(CO)_2_ under argon to afford 17-membered *m*-pyridinophanes **293a**,**b** and 18-membered parapyridinophanes **294a**,**b** in 10–15% isolated yield (Scheme 50).

**Scheme 51:**
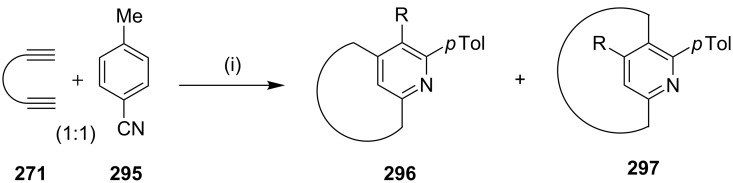
Synthesis of pyridinophanes **296** and **297** via a [2 + 2 + 2] cycloaddition. Reagents and conditions: (i) 15 mol % CpCo(CO)_2_, *o*-xylene (0.001 M), 140 °C, 100 h, 50–61%.

Maryanoff and co-workers [184] have reported the synthesis of various pyridinophanes by a [2 + 2 + 2] cycloaddition reaction mediated by a cobalt catalyst (Scheme 51). To this end, different bisalkynes **271** were reacted with *p*-toluenenitrile (**295**, 1 mol equiv) in 1:1 ratio to obtain [2,4]pyridinophane **296** and [2,5]pyridinophane **297** (Scheme 51).

**Scheme 52:**
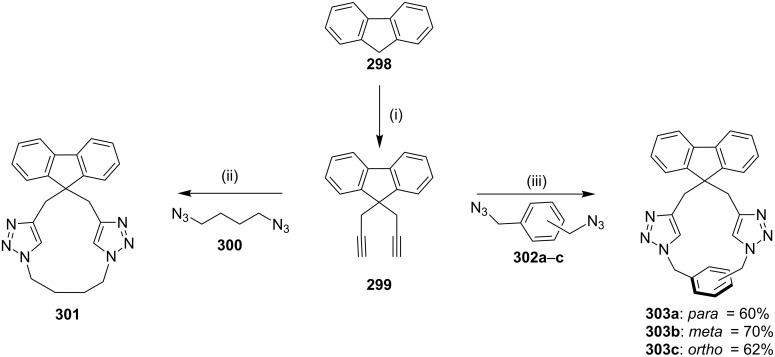
Synthesis of triazolophane by a 1,3-dipolar cycloaddition. Reagents and conditions*:* (i) propargyl bromide, NaOH, PhCH_2_N^+^Et_3_Cl, PhMe, 4 h, reflux 50%; (ii) **300**, CuSO_4_·5H_2_O/Na ascorbate, THF/H_2_O 1:1, 60 °C, 12 h, 42%; (iii) **302a–c**, CuSO_4_·5H_2_O/Na ascorbate, THF/H_2_O 1:1, 60 °C, 12 h, 60%.

**[3 + 2] Cycloaddition (1,3-dipolar cycloaddition/click reaction):** In 2010, Raghunathan and co-workers [185] have synthesized a *C*_2_-symmetric triazolophane by a copper(I)-catalyzed azide-alkyne cycloaddition, involving a click reaction. The dipropargyl fluorenyl derivative **299** was prepared from 9*H*-fluorene (**298**) and propargyl bromide, which on further treatment with 1,4-diazidobutane (**300**) and xylyl azides **302a–c** in the presence of CuSO_4_·5H_2_O and sodium ascorbate in THF/water (1:1) gave the corresponding macrocycles (**301**, 42%) and (**303a**–**c**, 60–70% yield, Scheme 52).

**Scheme 53:**
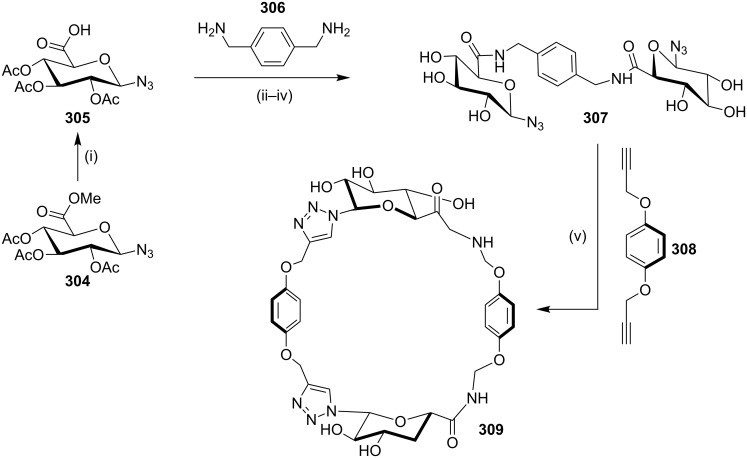
Synthesis of glycotriazolophane **309** by a click reaction. Reagents and conditions: (i) LiOH, H_2_O, MeOH, THF, 16 h, rt, 93%; (ii) ClCOCOCl, DMF (cat), CH_2_Cl_2_, rt, 0.5 h; (iii) **306**, DIPEA, 4 Å MS, CH_2_Cl_2_, 0 °C, 2 h; (iv) NaOMe, MeOH/CH_2_Cl_2_, rt, 3 h, (three steps 37%); (v) **308**, CuSO_4_, sodium ascorbate, MeCN/H_2_O, rt, 13 h, 56%.

Murphy and Leyden [186] have reported the synthesis of a glycotriazolophane **309** (carbohydrate–triazole–cyclophane hybrid) from a sugar amino acid via a copper-catalyzed azide-alkyne cycloaddition sequence. An aminosugar acid was identified as a useful building block to generate cyclophanes. Thus, the treatment of **304** with oxalyl chloride in the presence of DMF generated the acid chloride, which on further reaction with *p*-xylylenediamine (**306**) in the presence of *N*,*N’*-diisopropylethylamine (DIPEA) in dichloromethane followed by de-*O*-acetylation gave the bisazide **307** (37%). The latter compound was reacted with the dialkyne **308** in the presence of CuSO_4_ and sodium ascorbate in acetonitrile/water to deliver the desired cyclophane derivative **309** (56%, Scheme 53).

**Figure 11:**
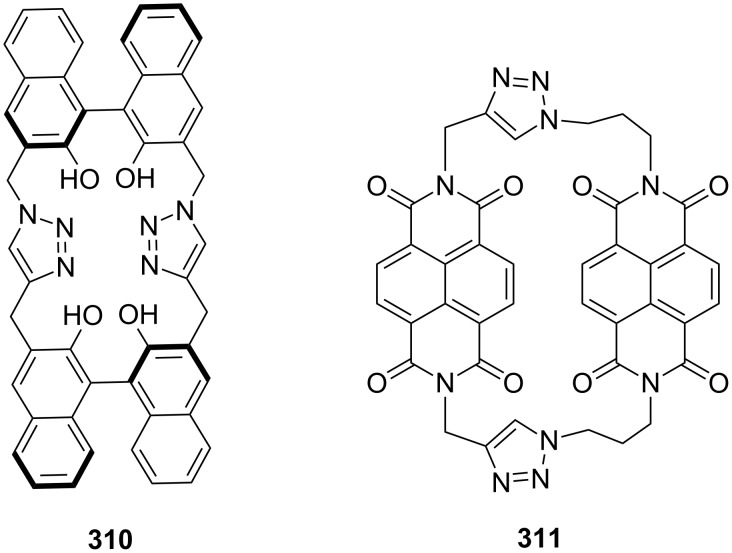
Cyclophanes **310** and **311** prepared via click chemistry.

Similarly, a novel BINOL-based cyclophane **310** has been synthesized via click chemistry by incorporating two triazole moieties in the macrocycle [187]. Li and co-workers [188] have reported the synthesis of the naphthalene-diimide-based cyclophane **311** for understanding supramolecular interactions by metal ions (Figure 11).

**Scheme 54:**
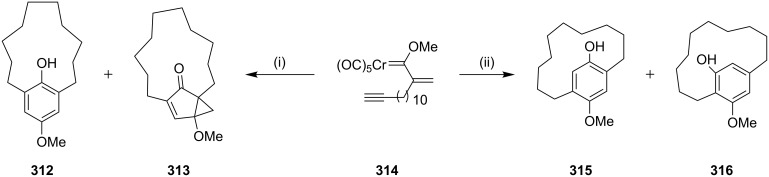
Synthesis of cyclophane via the Dötz benzannulation. Reagents and conditions: (i) THF, 100 °C, 12 h, (**312**, 15%), (**313**, 42%); (ii) benzene, 12 h, 100 °C, (**315**, 40%), (**316**, 21%).

**[3 + 2 + 1] Cycloaddition (Dötz benzannulation):** In 2003, Wulff and co-workers [189] synthesized cyclophane derivatives using the Dötz benzannulation as a key step. They found that the Fischer carbene complex **314** in a coordinating solvent such as THF lead to the products **312** (15%) and **313** (42%) whereas a non-coordinating solvent like benzene delivered products **315** (40%) and **316** (21%, Scheme 54).

**Scheme 55:**
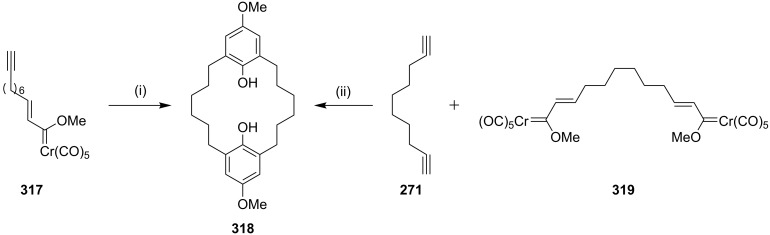
Synthesis of [6,6]metacyclophane by a Dötz benzannulation. Reagents and conditions: (i) THF, 100 °C, 14 h, (**318**, 39%); (ii) THF, 100 °C, 14 h, (**318**, 31%).

Wulff and Wang [190] have synthesized [6,6]metacyclophane via an intermolecular benzannulation reaction of Fischer carbene complexes with a residual alkyne to generate the 18-membered ring. Two molecules of the Fischer carbene complex **317** reacted by an intermolecular fashion to generate the [6,6]metacyclophane **318** (39%). Alternatively, a double benzannulation of a biscarbene complex **319** with 1,9-decadiyne (**271**) delivered [6,6]metacyclophane **318** (31%) (Scheme 55).

**Scheme 56:**
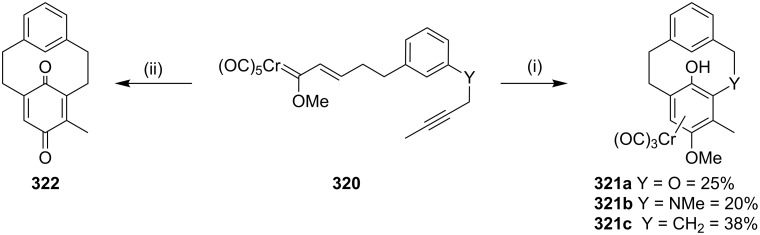
Synthesis of cyclophanes by a Dötz benzannulation. Reagents and conditions: (i) THF, 65 °C, 3 h; (ii) (*n*-Bu)_2_O, 90 °C, 2 h, CAN, (**321**, 40%).

Dötz and Gerhardt [191] have synthesized the [2,2]metacyclophane via chromium-mediated intermolecular benzannulation. In this connection, methoxy(alkynyl)carbene complex undergo an intramolecular benzannulation reaction in the presence of a polar solvent such as THF to deliver [6,6]metacyclophane (**321a**, 25%, **321b**, 20% and **321c**, 38%). Similarly metabenzoquinonophane **322** has been synthesized starting with **320** by an in situ oxidation of the benzannulated product by using cerium(IV) ammonium nitrate (40%, Scheme 56).

**Scheme 57:**
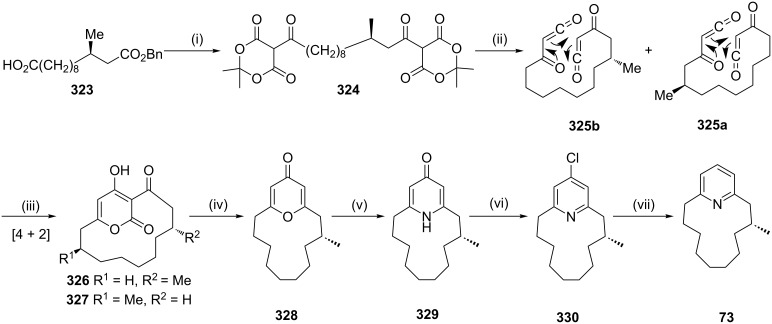
Synthesis of muscopyridine (**73**) via an intramolecular DA reaction of ketene. Reagents and conditions: (i) (a) SOCl_2_, reflux, 30 min; (b) Meldrum’s acid, DMAP, CH_2_Cl_2_, 0 °C, 2 h, then rt, 1 h; (ii) Ph-Cl, reflux, 20 h, 84%; (iii) heating; (iv) conc. HCl, reflux, 12 h, 89%; (v) NH_3_, EtOH, sealed tube, 140 °C, 72 h, 87%; (vi) POCl_3_, reflux, 1 h, 93%; (vii) H_2_, Pd/C, AcONa, rt, 12 h, 89%.

**Intramolecular Diels–Alder (DA) reaction:** Suwa and co-workers [192] have synthesized the muscopyridine by a [4 + 2] cycloaddition of the bisketene **325**. The condensation of acid dichloride derived from **323** with two molecules of Meldrum’s acid gave **324** which on thermal activation in chlorobenzene yielded bisketenes **325a** and **325b**. These two ketene derivatives underwent an intramolecular cycloaddition to afford a 1:1 mixture of **326** and **327** (96%, Scheme 57). On heating with concentrated HCl, **326** and **327** were transformed to pyrone derivative **328** (89%). A solution of the compound **328** in ethanol saturated with ammonia was heated in a stainless sealed tube for 3 days to deliver the pyridinone derivative **329** (87%). Further, chlorination of the pyridinone **329** afforded the chloropyridine **330** (93%). Subsequently, hydrogenolysis of the pyridine derivative **330** gave the target muscopyridine (**73**, 89%).

**Scheme 58:**
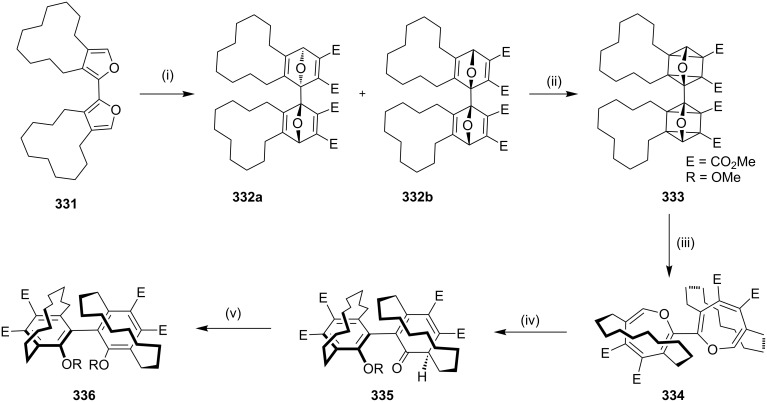
Synthesis of bis[10]paracyclophane **336** via Diels–Alder reaction. Reagents and conditions: (i) DMAD, PhMe, reflux, 4 h, 77%; (ii) Et_2_O, *h*v, 2 h, 78%; (iii) THF, reflux, 2 h, 81%; (iv) CF_3_OAc, CH_2_Cl_2_, rt, 24 h, 37%; (v) CH_2_Cl_2_, *t*-BuOK, methyl triflate, rt, 2 h, 63%.

**[4** + **2] Cycloaddition (Diels–Alder reaction):** In 2003, Tochtermann and co-workers [193] have synthesized a bis[10]paracyclophane with two chiral planes and one chiral axis via the DA reaction as a key step. The bifuran derivative **331** was subjected to a DA sequence with dimethyl acetylenedicarboxylate (DMAD) to deliver compounds **332a**,**b** (77%). These DA adducts were irradiated in diethyl ether/dichloromethane (5:1) to offer the corresponding bioxaquadricyclane **333**, subsequent thermolysis gave the bioxepine **334** (81%). Finally, aromatization of bioxepine **334** with trifluoroacetic acid (TFA) delivered ketophenol **335** (37%), which on further treatment with potassium *tert*-butoxide/methyltriflate mixture, gave the dimethyl ether bis[10]paracyclophane **336** (63%, Scheme 58).

**Scheme 59:**
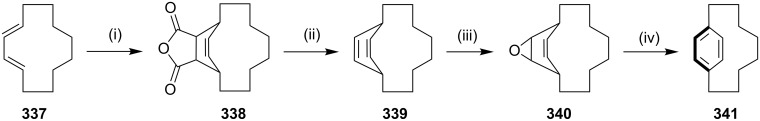
Synthesis of [8]paracyclophane via DA reaction. Reagents and conditions: (i) maleic anhydride, 3–5 h, 21%; (i) Pb(OAc)_4_, PhMe/pyridine, 2 h, 21%; (iii) *m*CPBA, 80%; (iv) HCl, 93%.

In 1980, Gassman and co-workers [194] have synthesized [8]paracyclophane via the DA reaction as a key step. In this connection, 1,3-cyclododecadiene (**337**) was reacted with maleic anhydride to give the DA product **338** (21%). Later, the DA adduct **338** was heated under reflux in 10% aq tetrahydrofuran to afford the diacid, which on decarboxylation in the presence of lead tetraacetate in a toluene/pyridine mixture delivered compound **339** (22%). Treatment of **339** with 1 equiv of *m*-chloroperbenzoic acid gave the epoxide **340** (80%), followed by HCl treatment gave [8]paracyclophane **341** (93%) (Scheme 59).

**Synthesis of the macrocyclic portion of longithorone C (DA reaction):** In 1994 longithorone A was first described by Schmitz and co-workers [195]. This unusual heptacyclic marine natural product is a cytotoxic agent. Its synthesis is considered difficult due to the stereocenters present in the ring system of longithorone A and E. Moreover, hindered rotation around the quinone moiety adds even more complexity to its synthesis.

**Scheme 60:**
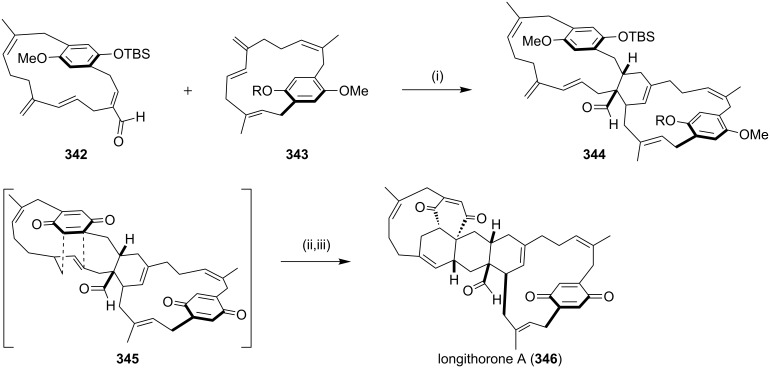
Biomimetic synthesis of (−)-longithorone A. Reagents and conditions: (i) Me_2_AlCl, CH_2_Cl_2_, −20 °C, 70%, 1:1.4 diastereomers; (ii) TBAF, THF, 0 °C; (iii) PhI(O), MeCN/H_2_O, 0–25 °C, 90% two steps.

Recently, Shair and co-workers [196] have reported the enantioselective synthesis of (−)-longithorone A by using a conventional synthesis to realize the proposed biosynthesis, which was put forward by Schmitz involving an intermolecular and an intramolecular DA reaction of two [12]paracyclophanequinone [197]. Based on this proposal Shair and co-workers attempted the synthesis of the natural product (−)-longithorone A. Diene **343** and the dienophile **342** were synthesized by several steps and subsequently subjected to the DA sequence to afford the rigid (−)-longithorone A (**346**, 90%, Scheme 60).

**Scheme 61:**
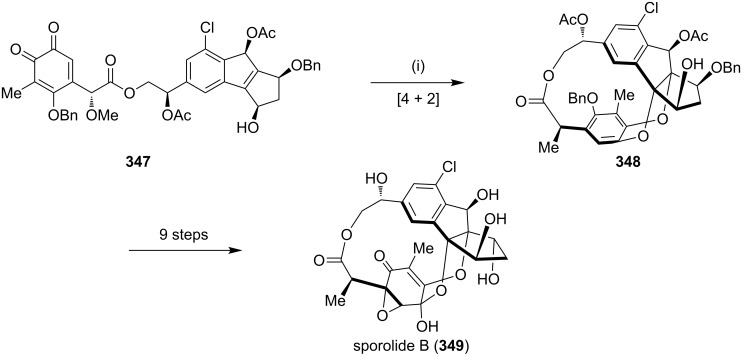
Synthesis of sporolide B (**349**) via a [4 + 2] cycloaddition reaction. Reagents and conditions: (i) PhMe, 110 °C, 1.5 h, 40% (based on 50% recovered starting material).

Nicolaou and co-workers [198] have reported the synthesis of sporolide B (**349**). The synthesis involves a DA reaction between *o*-quinone as the diene component and indene derivatives as dienophiles. This total synthesis also involves a Ru-catalyzed [4 + 2] cycloaddition reaction to generate a highly substituted indene system containing a chlorine substituent on the aromatic ring (Scheme 61).

**Scheme 62:**
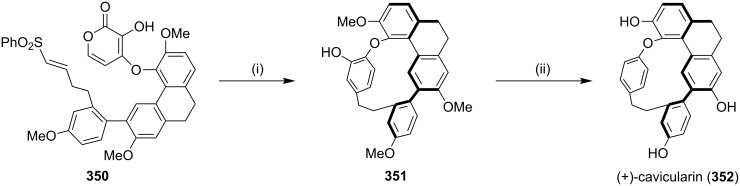
Synthesis of the framework of (+)-cavicularin (**352**) via a [4 + 2] cycloaddition. Reagents and conditions: (i) cinchona alkaloid derivative, EtOAc, 3 Å MS, 45 °C; (ii) (a) Tf_2_O, CH_2_Cl_2_, 0 °C, 45% (2 steps); (b) NH_4_CO_2_H, Pd/C, MeOH, 70 °C, quant.; (c) BBr_3_, CH_2_Cl_2_, 80%.

Cavicularin, a natural product containing a cyclophane system was isolated from the liverwort *Cavicularia densa.* Among several approaches to prepare this natural product, Beaudry and Zhao [199] have reported the synthesis of the basic architecture of (+)-cavicularin (**352**) by using the DA reaction of pyrone and vinyl sulfone (Scheme 62). They have reported the first intramolecular enantioselective DA reaction of the α-pyrone, also regioselective one-pot three-component Suzuki reaction of a dibromoarene to form a highly substituted terphenyl system (Scheme 62).

#### Rearrangement reactions

**Scheme 63:**
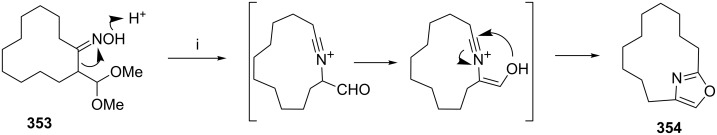
Synthesis of oxazole-containing cyclophane **354** via Beckmann rearrangement. Reagents and conditions: (i) polyphosphoric acid, toluene, reflux, overnight, 46%.

**Beckmann rearrangement:** Uemura and coworkers [200] have synthesized the cyclophane-containing oxazole moiety via a Beckmann rearrangement as a key step. α-Formylketoxime dimethyl acetal **353** was synthesized in several steps and subjected to a Beckmann rearrangement by using polyphosphoric acid in toluene heated under reflux conditions to give oxazole-based cyclophane **354** in 46% (Scheme 63).

**Scheme 64:**
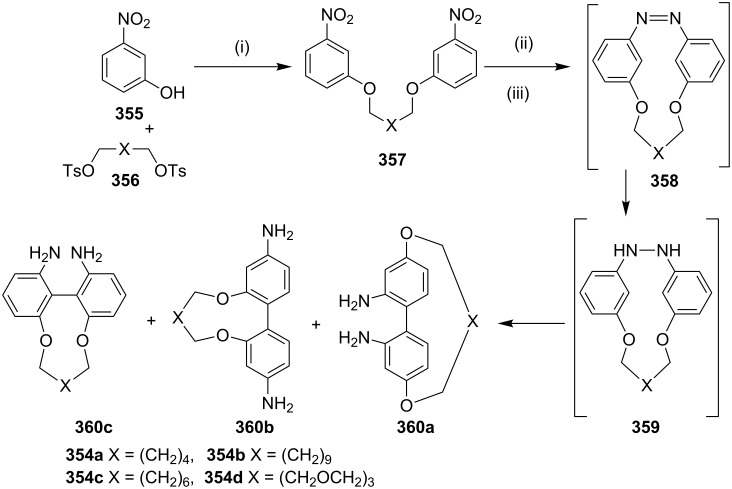
Synthesis of cyclophanes **360a–c** via benzidine rearrangement. Reagents and conditions: (i) **356a**–**d**, K_2_CO_3_, DMF; (ii) Zn, NaOH; (iii) HCl.

**Benzidine rearrangement:** Benniston and co-workers [201] have reported the synthesis of cyclophanes **360a–c** involving a benzidine rearrangement [202–208]. The *m*-nitrophenol (**355**) was reacted with ditosylate **356** to generate *m*-nitrophenol ether derivative **357**, which on a reduction with Zn in MeOH gave azo-derivative **358**. It was further converted into the hydrazo compound **359** which underwent a benzidine rearrangement under acidic conditions to deliver cyclophanes **360a–c**. The cyclophanes obtained here involve the migration of nitrogen on the aromatic ring (Scheme 64).

**Scheme 65:**
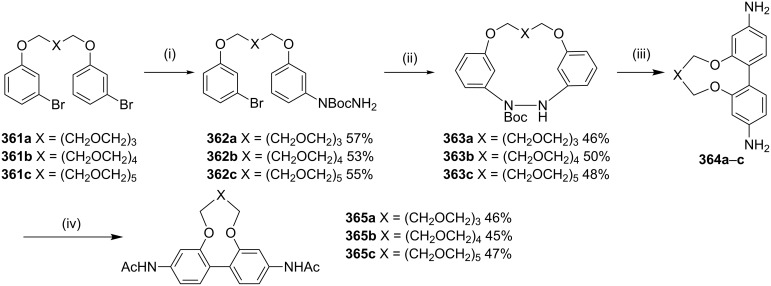
Synthesis of cyclophanes **365a–c** via benzidine rearrangement. Reagents and conditions: (i) BocNHNH_2_, CuI, Cs_2_CO_3_, 1,10-phen, DMF, 80 °C, 24 h; (ii) Pd(OAc)_2_, P(*t*-Bu)_3_, PhMe, 110 °C, 12 h; (iii) aq HCl, EtOH, 80 °C, reflux, 2 h; (iv) AcCl, NaOAc, MeCN, rt, 12 h.

Cho and co-workers [209] have reported the synthesis of 4,4-diaminobiphenyls (benzidine) connected with a polyether unit at the 2,2'-positions using the benzidine rearrangement. The cyclophane synthesis of **365** starts with the preparation of **361a–c** starting with *m*-bromophenol and polyether ditosylates. The Cu(I)-catalyzed coupling reactions of the bis(*m*-bromophenyl) ethers **361a–c** provided the monohydrazides **362a–c** (53–57%). Cyclization reactions were carried out by using a Pd catalyst delivering diarylhydrazides **363a–c** (46–50%). Later, the hydrazides **363a–c** were heated in EtOH with a catalytic amount of aq HCl to generate the corresponding benzidines **364a–c**, as indicated by their crude ^1^H NMR spectra. These products were subjected to an acetylation sequence to generate the cyclophane-based acetamides **365a–c** (Scheme 65).

**Scheme 66:**
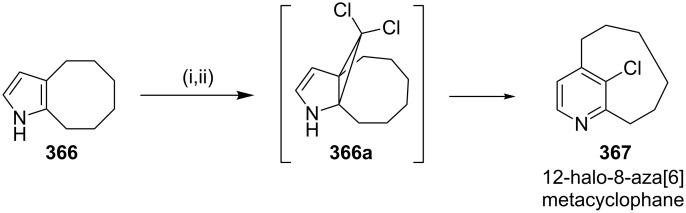
Synthesis of metacyclophane **367** via Ciamician–Dennstedt rearrangement. Reagents and conditions: (i) Cl_3_CCO_2_Na (5 equiv), 1,2-dimethoxyethane, reflux, 4 h; (ii) Hg(Ph)(CBr_3_) (2 equiv), benzene, reflux, 24 h.

**Ciamician–Dennstedt rearrangement:** Reese and Dhanak [210] have synthesized a strained cyclophane such as [6](2,4)pyridinophane derivatives **367** by using a ring expansion strategy. Here, pyrrole derivative **366** was treated with dihalocarbene giving the cyclopropane intermediate **366a** which was further converted into pyridinophane **367** by a ring expansion (Scheme 66).

**Scheme 67:**
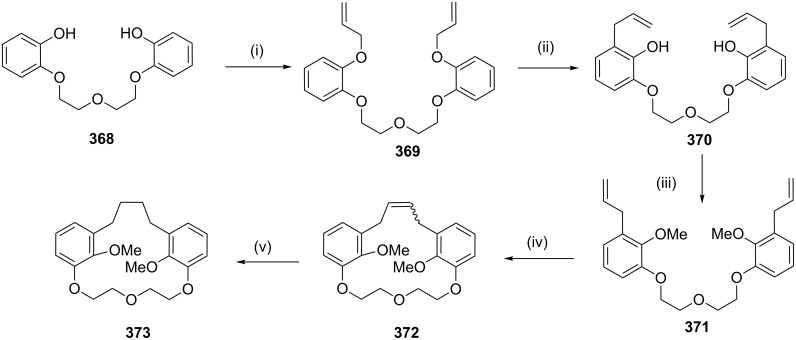
Synthesis of cyclophane by tandem Claisen rearrangement and RCM as key steps. Reagents and conditions: (i) allyl bromide, acetone, reflux, 12 h, 92%; (ii) dichlorobenzene, reflux, 24 h, 64%; (iii) MeI, K_2_CO_3_, acetone, reflux, 6 h, 88%; (iv) G-I (**12**), PhMe, reflux, 12 h, 56%; (v) H_2_, Pd/C, EtOAc, 12 h, rt, 98%.

**Claisen rearrangement:** To develop new strategies to diverse cyclophanes, Kotha and Waghule [211] have reported the synthesis of cyclophane **373** by using the double Claisen rearrangement and an RCM as key steps. Bisphenol **368** was converted to *o*-allyl derivative **369**, which on a Claisen rearrangement followed by protection of the phenolic hydroxy groups gave **371**. An RCM of **371** followed by the hydrogenation of the RCM product **372** gave cyclophane **373** (Scheme 67). By using a similar approach various cyclophanes were synthesized starting with resorcinol as well as hydroquinone and attaching an ethyleneoxy chain of different length (Scheme 67) [212].

**Scheme 68:**
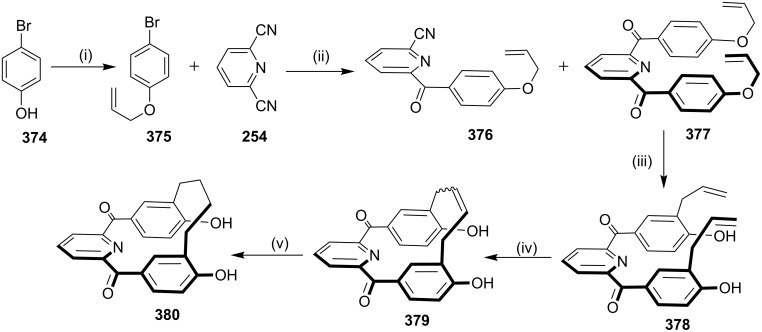
Synthesis of cyclophane derivative **380**. Reagents and conditions: (i) K_2_CO_3_, CH_3_CN, allyl bromide, rt, 6 h, 98%; (ii) Mg, I_2_, THF, rt, 12 h, 59%; (iii) 1,2-dichlorobenzene, 8 h, 190 °C, 81%; (iv) G-II (**13**, 5 mol %), PhMe, reflux, 18 h, 62%; (v) Pd/C, MeOH, rt, 12 h, 81%.

Kotha and Shirbhate [213] have synthesized the cyclophane derivative **380**. Commercially available 4-bromophenol (**374**) and allyl bromide were reacted in the presence of a mild base such as K_2_CO_3_ to generate *O*-allyl derivative **375** (98%). Later, commercially available 2,6-pyridinedicarbonitrile (**254**) was reacted with the Grignard reagent prepared from *O*-allylbromophenol (**375**), activated magnesium turnings, and iodine (for activation) in THF. The desired bis-*O*-allyl derivative **377** was then directly subjected to a Claisen rearrangement at 180 °C in *o*-dichlorobenzene (*o*-DCB) for 8 h (Scheme 68). The diallylated compound **378** was subjected to RCM by using G-II (**13**) as a catalyst to generate the desired cyclophane **379** (62%) as a 1:1 mixture of *cis* and *trans*-isomers. However, the *trans*-isomer of RCM product **379** was crystallized in methanol and acetonitrile (1:1) after several attempts (Scheme 68).

**Scheme 69:**
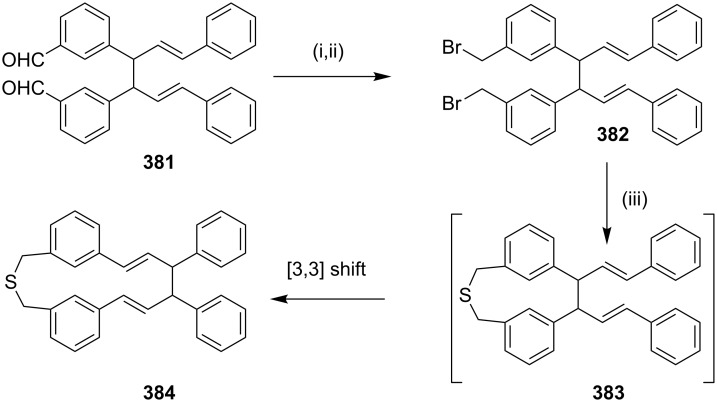
Synthesis of metacyclophane via Cope rearrangement. Reagents and conditions: (i) MeOH, NaBH_4_, rt, 1 h, 95%; (ii) PBr_3_, C_6_H_5_CH_3_, Et_2_O, 12 h, 60 °C, 96%; (iii) Na_2_S, C_6_H_6_, EtOH, Cs_2_CO_3_, 8 h, 80 °C, 51%.

**Cope rearrangement:** In 1986, Vögtle and Eisen [214] have succeeded in assembling a tetraarylbiallyl skeleton by doubly bridged metacyclophane derivatives, which underwent a spontaneous Cope rearrangement under mild reaction conditions. Tetraaryl dialdehyde **381** was prepared in several steps and further reduction of the aldehyde functionality with NaBH_4_ in methanol gave the diol. Bromination of the diol with PBr_3_ gave the dibromotetraaryl derivative **382** (75%). Subsequently, cyclization of the bisbromide **382** gave the product **384** through a [3,3]-sigmatropic rearrangement (51%, Scheme 69).

**Scheme 70:**
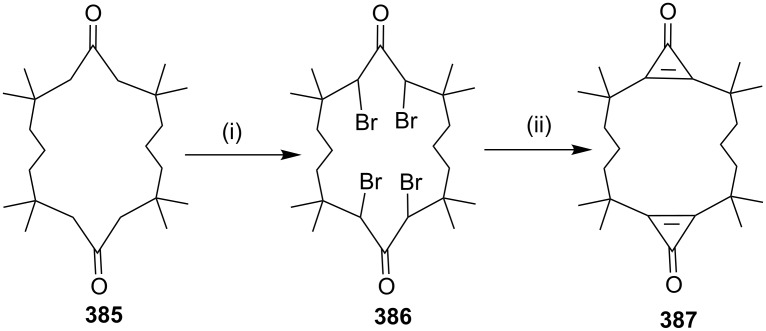
Synthesis of cyclopropanophane via Favorskii rearrangement. Reagents and conditions: (i) Br_2_, CH_2_Cl_2_, 5 h, rt, 46%; (ii) KO*t*-Bu, THF, −40 °C, 30 min, 27%.

**Favorskii rearrangement:** In 2005, Gleiter and co-workers [215] have synthesized sterically stabilized cyclopropanophanes, containing non-benzenoid three-membered aromatic rings. Diketone **385** was subjected to bromination in the presence of bromine which afforded tetrabromide **386** with *anti*-orientation to the keto group with four equatorial bromine atoms (46%). Subsequently, tetrabromo derivative **386** was converted to cyclopropanophane **387** (27%) by Favorskii rearrangement and thus generated the three-membered ring systems (Scheme 70).

**Scheme 71:**
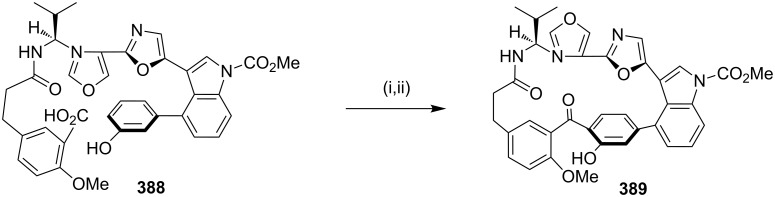
Cyclophane **389** synthesis via photo-Fries rearrangement. Reagents and conditions: (i) DMAP, EDCl/CHCl_3_, (0.004 M), 66%; (ii) *h*v, benzene (0.001 M), 23 °C, 76%.

**Photo-Fries rearrangement**: It was shown that Diazonamide has potent in vitro activity against HCT-116 human colon carcinoma and B-16 murine melanoma cancer cells and several attempts have been reported to synthesize this alkaloid. Magnus and Lescop have reported [216] the synthesis of the diazonamide core **388** by using a photo-Fries rearrangement with the substrate **389** (Scheme 71).

**Scheme 72:**
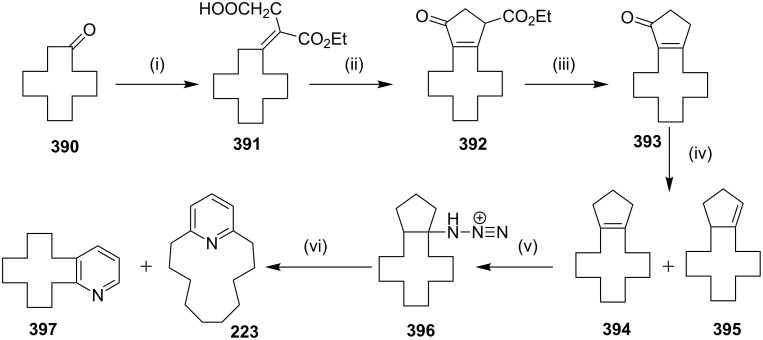
Synthesis of normuscopyridine (**223**) via Schmidt rearrangement. Reagents and conditions: (i) ethyl succinate, KO*t*-Bu, *t*-BuOH, reflux, 22 h, 84%; (ii) ZnCl_2_, PPA, 95 °C, 45 h; (iii) HCl, AcOH, reflux, 19 h, (2 steps 47%); (iv) Na, ethylene glycol, N_2_H_4_, reflux, 3 h, 55%; (v) CHCl_3_, EtOH, HN_3_, 30 min, 50 °C; (vi) 1-methylnaphthalene, 10% Pd/C, reflux, 3.5 h (**223**, 17% in 2 steps; **397**, 16% in 2 steps).

**Schmidt rearrangement:** The first approach described here involves the Stobbe condensation of cyclododecanone (**390**) with ethyl succinate to deliver carboxylic acid **391**, which on cyclization with zinc chloride in polyphosphoric acid gave cyclopentanone derivative **392**. Acidic hydrolysis of ester **392** and simultaneous decarboxylation gave the unsaturated ketone **393**. Wolff–Kishner reduction of the cyclopentenone derivative **393** gave the two isomeric olefins **394** and **395**. An application of the Schmidt reaction with a mixture of compounds **394** and **395** followed by dehydrogenation with Pd/C afforded [10](2,6)pyridinophane **223** and its 2,3-isomer **397** (Scheme 72) [217].

**Scheme 73:**
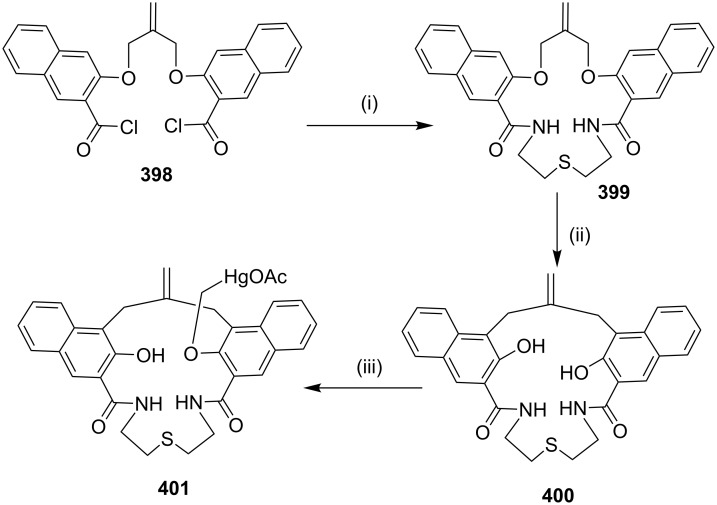
Synthesis of crownophanes by tandem Claisen rearrangement. Reagents and conditions: (i) diamine, Et_3_N, THF; (ii) NMP, reflux; (ii) Hg(OAc)_2_, DMF/ether.

**Tandem Claisen rearrangement**: In 2008, Hiratani and co-workers [218] have reported the synthesis of the sulfur-containing crownophane **401** by using the tandem Claisen rearrangement as a key step. Diacetyl chloride **398** was coupled with various sulfur-containing diamines followed by tandem Claisen rearrangement of the resulting exemplar amide derivative **399** in *N*-methyl-2-pyrrolidone (NMP) which yielded the desired sulfur-containing crownophane **400**. Later, the reaction of this crownophane **400** with Hg(OAc)_2_ gave the organomercurated dihydrobenzofuran containing macrocycle **401** (Scheme 73).

**Scheme 74:**
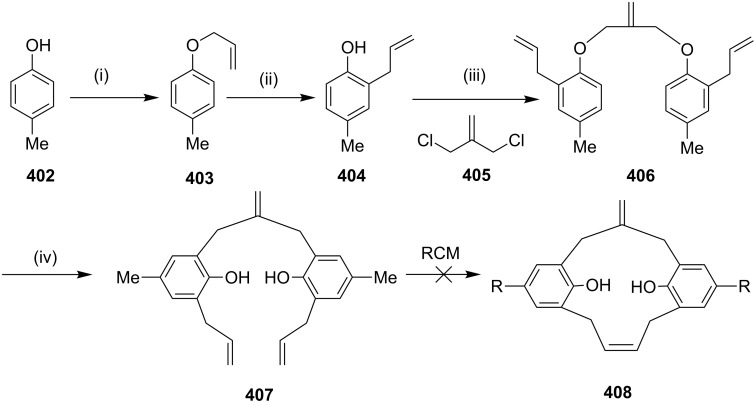
Attempted synthesis of cyclophanes via tandem Claisen rearrangement and RCM. Reagents and conditions: (i) allyl bromide, K_2_CO_3_, acetone, reflux, 16 h, 92%; (ii) 160–180 °C, 6 h, 77%; (iii) **405**, K_2_CO_3_, acetone, reflux, 6 h, 84%; (iv) BCl_3_, CH_2_Cl_2_, −60 °C to rt, 3 h, 27%.

Kotha and co-workwers [212] have also attempted the synthesis of cyclophane derivatives involving the tandem Claisen rearrangement and an RCM as key steps. To this end, *p*-cresol (**402**) was reacted with allyl bromide to give allyl ether **403**, which undergoes a Claisen rearrangement to deliver *O*-allylphenol derivative **404**. Phenol derivative **404** was reacted with 3-chloro-2-(chloromethyl)-1-propene (**405**) to generate the key precursor **406**. Tandem Claisen rearrangement of **406** in the presence of BCl_3_ yielded the rearranged product **407** (27%). Various attempts to generate the RCM product **408** from **407** or its derivatives were not successful (Scheme 74).

#### Alkylation

**Scheme 75:**
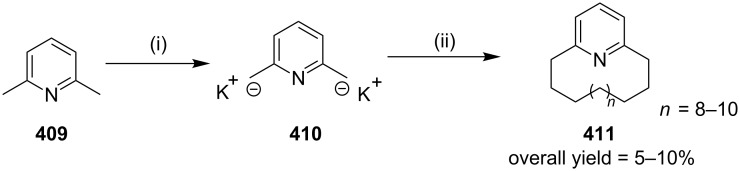
Synthesis of muscopyridine via alkylation with 2,6-dimethylpyridine anion. Reagents and conditions: (i) K*t-*OBu, *n*-BuLi, C_6_H_12_, reflux, 1 h, 100%; (ii) dibromoalkanes, THF, –78 °C to rt.

Bates and Ogle [219] have reported the synthesis of the normuscopyridine and its analogues by reacting the dipotassium salt of lutidine with dibromoalkanes. To this end, 2,6-dimethylpyridine (**409**) was treated with *n*-BuLi and KO*t*-Bu to generate dianion **410**, which on reaction with dibromoalkanes gave the symmetrical pyridinophanes **411** in 5–10% overall yield (Scheme 75).

#### Friedel–Crafts alkylation

**Scheme 76:**
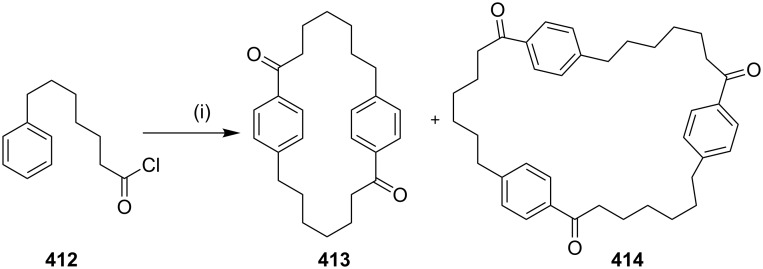
Synthesis of cyclophane via Friedel**–**Craft acylation. Reagents and conditions*:* (i) CS_2_, AlCl_3_, 7 d, rt, (**413**, 5%, **414**, 0.4%).

In 1954, Schubert and co-workers [220] have synthesized dimeric and trimeric benzocyclanone via Friedel–Crafts reaction as a key step. In this regard, compound 7-phenylheptanoyl chloride (**412**), was subjected to cyclization under high-dilution conditions to deliver dimer **413** (5%) and trimer **414** (0.4%, Scheme 76).

#### Friedel–Craft acylation

**Scheme 77:**
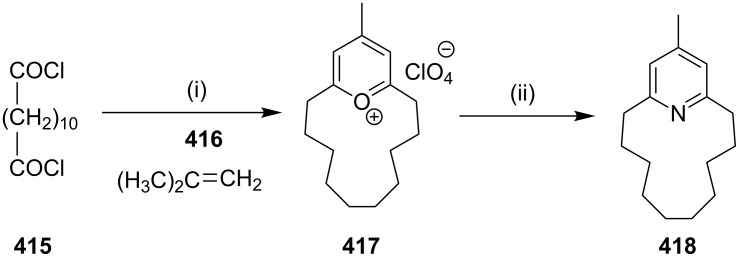
Pyridinophane **418** synthesis via Friedel–Craft acylation. Reagents and conditions: (i) **416**, AlCl_3_, CH_3_NO_2_, 50 °C, 8 h, 2%; (ii) liquid NH_3_/*t*-BuOH, 1%.

Georgi and Retey [221] have synthesized the isomer of muscopyridine **418** involving the pyrylium salt **417**. The overall yield of the reaction was low. Diacylation of isobutylene (**416**) with dichloride **415** in the presence of aluminum chloride gave pyrylium salt **417** which on further treatment with ammonia gave pyridinophane **418** in low yield (Scheme 77).

#### Kotha–Schölkopf reagent [222]

**Scheme 78:**
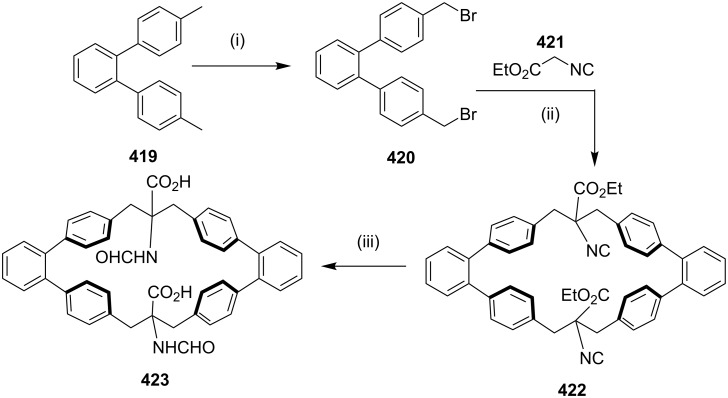
Cyclophane synthesis involving the Kotha–Schölkopf reagent **421**. Reagents and conditions: (i) NBS, AIBN, CCl_4_; (ii) BEMP, CH_3_CN, 0 °C; (iii) HCl.

Kotha and co-workers [223] have reported the first and unexpected synthesis of macrocyclic cyclophane containing the unusual amino acid derivative **423** by using phosphazene as a base without high-dilution conditions (Scheme 78). Coupling of the two bromo-substituted rings was carried out with ethyl isocyanoacetate (Kotha–Schölkopf reagent).

**Scheme 79:**
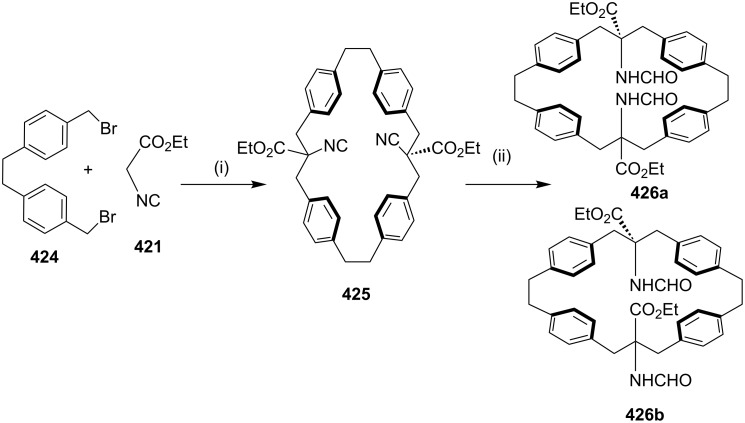
Cyclophane synthesis involving the Kotha–Schölkopf reagent **421**. Reagents and conditions*:* (i) BEMP, CH_3_CN, 0 °C; (ii) HCl.

They also reported the synthesis of macrocyclic cyclophane-based unusual α-amino acid (AAA) derivatives **426** involving ethyl isocyanoacetate (**421**) and 1,2-bis(4-(bromomethyl)phenyl)ethane under phase-transfer catalysis (PTC) conditions using a phosphazene base (BEMP). Out of two isomers formed, *trans*-isomer **426a** was crystallized from petroleum ether (Scheme 79) [224–225].

#### (*p*-Tolylsulfonyl)methyl isocyanide (TosMIC)

**Scheme 80:**
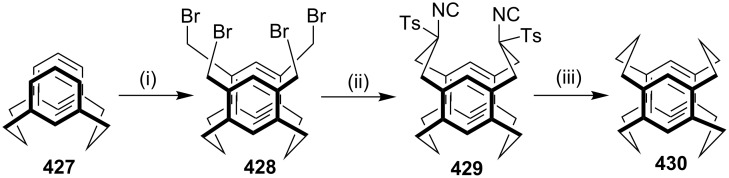
Cyclophane synthesis by coupling with TosMIC. Reagents and conditions: (i) (a) ClCH_2_OCH_3_, TiCl_4_, CS_2_; (b) NaBr, EtBr, DMF; (ii) NaH, TosMIC, DMF, rt.; (iii) Li, liquid NH_3_, EtOH.

Shinmyozu and co-workers [226] have reported the synthesis of [34](1,2,4,5)cyclophane **430** by using the TosMIC reagent. This reagent is useful to prepare barrelenophane which can be further converted into semibullvalenophane (Scheme 80).

#### Synthesis of azacyclophane via nitrobenzenesulfonyl (Ns)-amide method

**Scheme 81:**
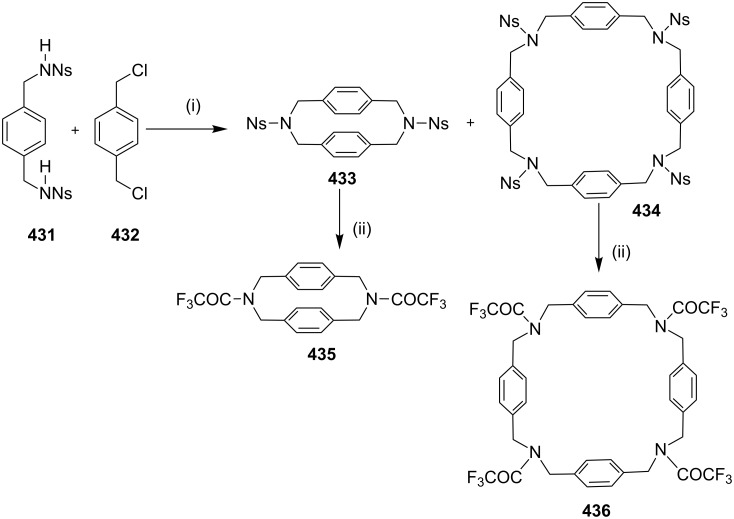
Synthesis of diaza[3_2_]cyclophanes and triaza[3_3_]cyclophanes. Reagents and conditions: (i) DMF, NaH, 6 h, 70 °C, 26%; (ii) EtSNa, DMSO, 50 °C, TFAA, Et_3_N, dioxane, rt, **435**, 26%, **436**, 5%.

In 2008, Okamoto and co-workers [227] have synthesized diaza[3_2_]cyclophanes and triaza[3_3_]cyclophanes. To this end, bis-Ns-amide **431** was prepared by several steps and it was further treated with NaH in DMF to generate the bis-amidate anion, which was coupled under high-dilution conditions with 1,4-bis(chloromethyl)benzene (**432**) at 70 °C to give the dimer **433** as well as the trimer **434**. Subsequently, deprotection of cyclophanes **433** and **434** was carried out with sodium ethanethiolate at 50 °C and the amino derivatives were acetylated with trifluroacetic anhydride to generate cyclophanes **435** (26%) and **436** (5%), respectively (Scheme 81).

#### Acyloin condensation

**Scheme 82:**
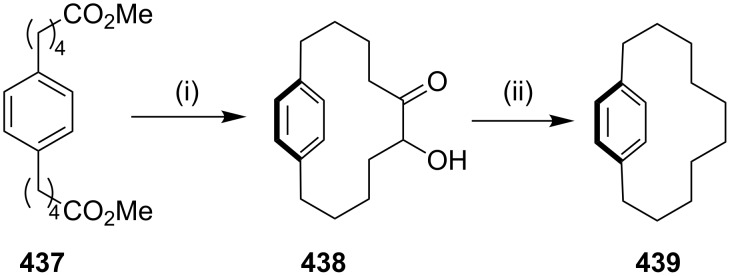
Synthesis of cyclophane **439** via acyloin condensation. Reagents and conditions: (i) Na, xylene, 75%; (ii) Zn, HCl/AcOH, 36%.

Rubin and coworkers [228] have synthesized cyclophane **439** by acyloin condensation. Furthermore, studies were carried out to find out the behavior of intramolecular energy transfer reaction (Scheme 82).

#### Aldol condensation

**Scheme 83:**
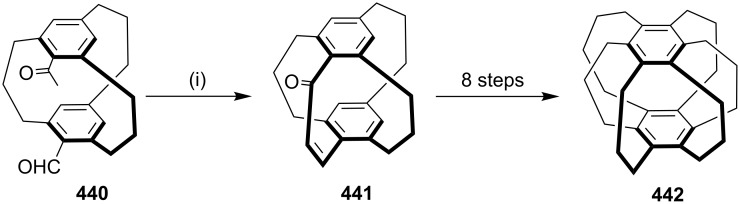
Synthesis of multibridged binuclear cyclophane **442** by aldol condensation. Reagents and conditions: (i) aq NaOH, THF, MeOH.

Shinmyozu and co-workers [229] have reported the synthesis of multibridged [3*_n_*]cyclophanes **442** by aldol condensation. Due to an enhanced transannular π–π interaction between two benzene rings and the hyperconjugation of the benzyl hydrogens with the benzene rings multibridged cyclophane **442** shows a high π-donating ability. Aldol condensation of ketoaldehyde **440** gave keto derivative **441** which was further extended to multibridged cyclophane **442** (Scheme 83).

#### Intramolecular esterification reaction

**Scheme 84:**
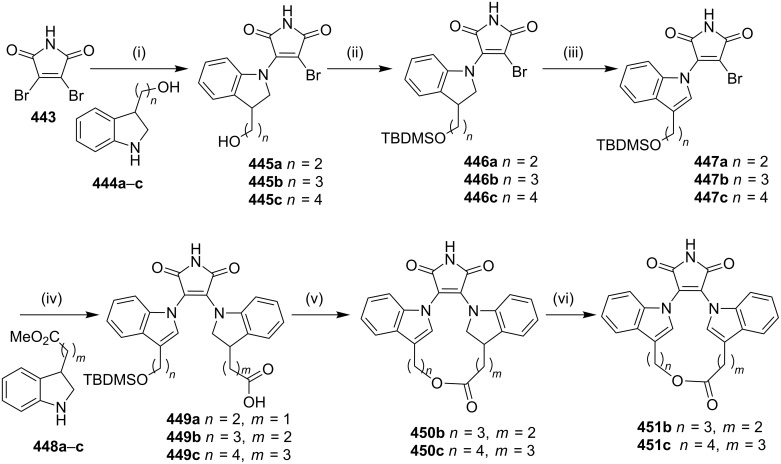
Synthesis of various macrolactones. Reagents and conditions: (i) iPr_2_EtN, DMF, 77–83%; (ii) TBDMSCl, imidazole, DMF, 12 h, rt, 10 h, 72–80%; (iii) DDQ, PhMe, 6 h, reflux, 72–75%; (iv) iPr_2_EtN, DMF, 12 h, rt, 51–63%; (v) *p*TSA, C_6_H_6_, reflux, 30 min, 15%; (vi) DDQ, PhMe, reflux, 3 h, 71–75%.

In 2014, Preobrazhenskaya and co-workers [230] have synthesized [15]-, [16]-, and [17]-membered lactones containing bis-3,4(indol-1-yl)maleimide framework via an intramolecular esterification reaction as a key step. 2,3-Dibromomaleimide (**443**) was coupled with various (2,3-dihydroindol-3-yl)alkanols (**444a–c**) in the presence of Et_3_N to give the corresponding ω-hydroxyalkyl derivatives **445a–c**. Next, the protection of the hydroxy groups with TBDMSCl led to the protected derivatives **446** (72–80%). The bromo derivatives **446** were subjected to dehydrogenation by 2,3-dichloro-5,6-dicyano-1,4-benzoquinone (DDQ) to obtain 3-bromo-4-[3-(ω-hydroxyalkyl)indol-1-yl])maleimides **447** (72–75%), which were further coupled with various (3,4-dihydroindol-3-yl)alkanoic acids **448** to deliver the bisindole derivatives **449**. The bisindoles **449** were then treated with a catalytic amount of *p*-toluenesulfonic acid in benzene and heated under reflux to afford the macrolactones **450**. Dehydrogenation by using DDQ oxidation gave various macrolactones **451** (68–75%, Scheme 84).

#### Yamaguchi esterification

**Scheme 85:**
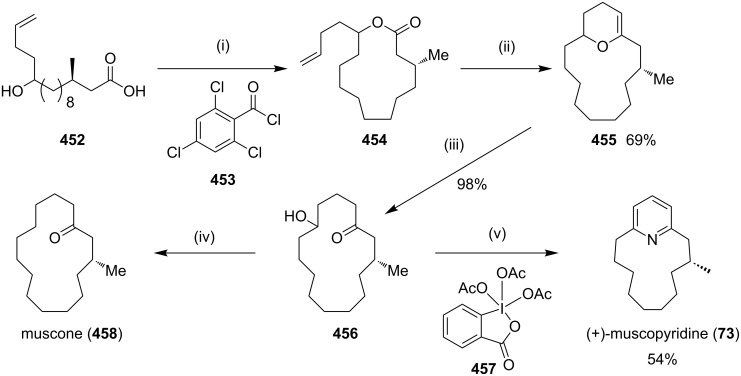
Synthesis of muscone and muscopyridine via Yamaguchi esterification. Reagents and conditions: (i) **453**, THF, PhMe, NEt_3_, DMAP, 45 °C, 6 h, 90%; (ii) TiCl_4_, Zn, TMEDA, PbCl_2_, CH_3_CHBr_2_, THF, 0 °C to rt, 2 h, 69%; (iii) SiO_2_, CH_2_Cl_2_, 48 °C, 2 h, 98%; (iv) (a) PhOC(S)Cl, DMAP, pyridine, CH_2_Cl_2_, 0 °C to rt, 10 h, 49%; (b) Bu_3_SnH, AIBN, PhH, reflux, 4 h, 97%; (v) **454**, NH_4_OH, EtOH, 160 °C, 18.5 h, 54%.

Rohanna and Rainier [231] have reported the synthesis of muscopyridine (**73**) by using an olefin lactone cyclization strategy. The Yamaguchi esterification of acid derivative **452** gave lactone **454**. Cyclization of lactone **454** yielded macrocyclic dihydropyran **455**. Silica gel mediated hydrolysis of the enol ether gave hydroxy ketone **456**, which served as a useful precursor to both muscone (**458**) and muscopyridine (**73**). Muscopyridine (**73**) has been generated via oxidation of the secondary alcohol **456**, followed by treatment of the 1,5-diketone with NH_4_OH. Alternatively, (*R*)**-**(**−**)**-**muscone (**458**) has been obtained from hydroxy ketone **456** by using the Barton–McCombie deoxygenation conditions (Scheme 85).

#### Elimination reactions

**Scheme 86:**
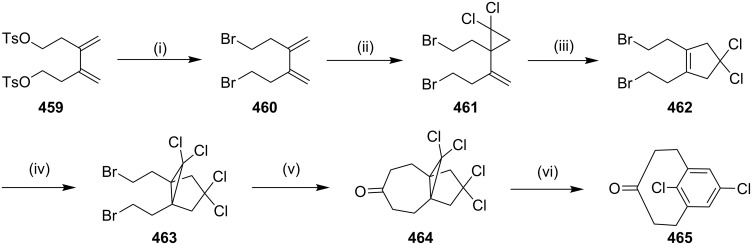
Synthesis of [5]metacyclophane via a double elimination reaction. Reagents and conditions: (i) LiBr, acetone, 12 h, reflux, 81%; (ii) CHCl_3_, *t*-BuOK, C_6_H_6_, rt, 80%; (ii) FVP, 480 °C, 5 × 10^−5^ mbar, 90%; (iv) CHCl_3_, PTC, NaOH (50%), 83%; (v) NaH, TosMIC, DMSO/Et_2_O, 15%; (vi) AgClO_4_/lutidine, THF, 90%.

**Double elimination reaction:** In 2001, Bickelhaupt and co-workers [232] have synthesized a [5]metacyclophane derivative with an sp^2^-center embedded at the central position of the bridge. Ditosylate **459** was converted to dibromide **460** by treatment with LiBr followed by the addition of dichlorocarbene to give the cyclopropane derivative **461** according to the Skattebøl method [233]. Next, it was rearranged to cyclopentane derivative **462** by using flash vacuum pyrolysis (FVP) [234]. The addition of dichlorocarbene to compound **462** by the method of Makosza [235] gave compound **463** which was cyclized with TosMIC [236–237] to generate propellane derivative **464**. Finally, the cyclophane **465** was obtained (70%) from **464** by a double elimination reaction by using AgClO_4_ and lutidine in THF (Scheme 86).

**Figure 12:**
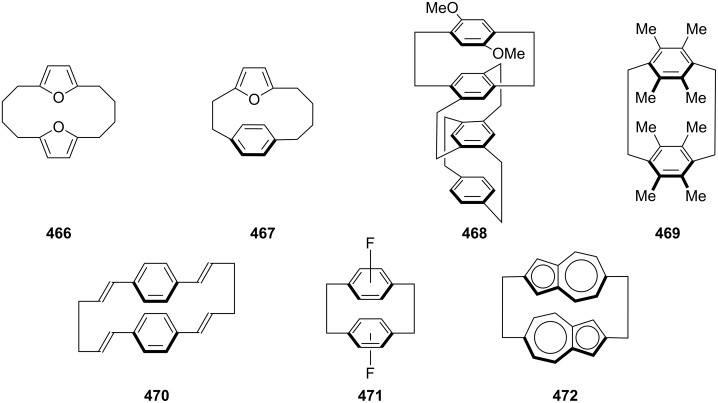
Cyclophanes **466–472** synthesized via Hofmann elimination.

**Hofmann elimination reaction:** The Hofmann elimination [238–241] is also named the Hofmann degradation. This reaction involves the elimination of alkyltrimethylamines and the product formation proceeds with an *anti*-stereochemistry. This reaction is generally suitable for assembling alkenes with one or two substituents. A general procedure involves the conversion of an amine into a tertiary amine followed by the treatment with an excess amount of methyl iodide. Further treatment with silver oxide, water and heating finally generates the alkene. The least substituted alkene is formed as a major product which is also known as the Hofmann rule [242–243]. The Hofmann elimination reaction is a classical and useful method to generate cyclophanes by cyclization of the obtained alkene compounds. Using this method a variety of cyclophanes have been prepared, including 1,6(2,5)-difuranacyclodecaphane (**466**) [244], paracyclo[2](2,5)-furanophane (**467**) [244], and quadrapole-layered paracyclophane **468** having charge-transfer properties [245]. Other examples of cyclophanes such as octamethyl[2.2]paracyclophane (**469**) [246–247], (2*E*,6*E*,9*E*,13*E*)-1,8(1,4)-dibenzenacyclotetradecaphane-2,6,9,13-tetraene (**470**) [248], difluoro[2,2]paracylophane (**471**) [249], and 2,6-azulylene (**472**) [250] are shown in Figure 12.

#### Baylis–Hillman reaction

**Scheme 87:**
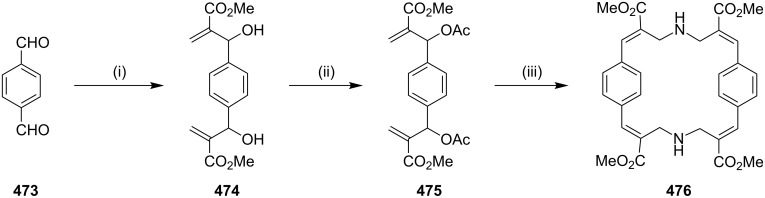
Synthesis of cryptophane via Baylis–Hillman reaction. Reagents and conditions: (i) methyl acrylate, DABCO, CH_3_CN, for 1–14 days, rt; (ii) CH_3_COCl, Et_3_N, THF, 5 h, rt, 97%; (iii) 8 M, NH_3,_ MeOH, 40 min, 28%.

In 1994, Foucaud and co-workers [251] have synthesized a macrocyclic cryptophane based on the Baylis–Hillman reaction. Dialdehyde **473** was reacted with methyl acrylate in the presence of diazabicyclooctane (DABCO) for 14 days at room temperature which resulted in the formation of diol **474**. Diol **474** was then subjected to an acetylation in the presence of AcOH to obtain allylic acetate **475** (97%). Finally, diacetate **475** was subjected to a nucleophilic substitution reaction by using ammonia in methanol to generate cryptophane **476** (28%, Scheme 87).

#### Double Chichibabin reaction

**Scheme 88:**
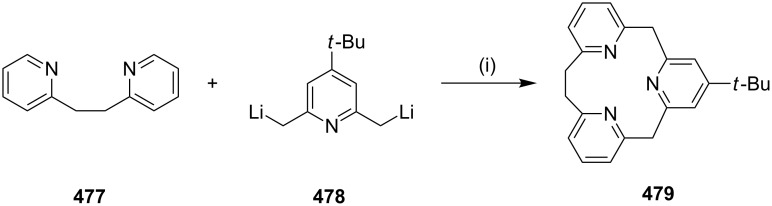
Synthesis of cyclophane **479** via double Chichibabin reaction. Reagents and conditions: (i) excess **478**, MeOH/H_2_O, 170 °C, 24 h, 60%.

The Chichibabin reaction is one of the most elegant reactions to synthesize 2-substituted aminopyridines. Caulton and co-workers [252] have reported the synthesis of [2.*n*.1](2,6)pyridinophane **479** by double Chichibabin reaction starting with **477** (Scheme 88). Also, using ab initio and DFT calculations, they reported new macrocyclic ligands to achieve an “intermediate” degree of stability and reactivity of d6 metal alkyl hydrido complexes.

**Scheme 89:**
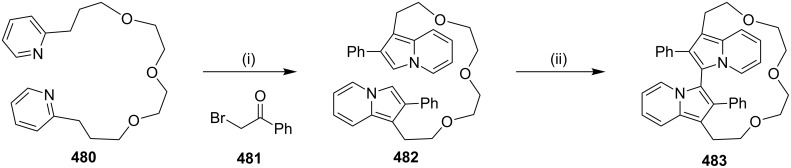
Synthesis of cyclophane **483** via double Chichibabin reaction. Reagents and conditions: (i) **481**, OH^−^; (ii) K_3_[Fe(CN)_6_].

Zabel and co-workers [253] have reported the synthesis of 3,3-biindolizine-based cyclophane **483** via Chichibabin reaction as a key step. Compound **480** was reacted with ω-bromoacetophenone (**481**) by adopting standard Chichibabin reaction conditions to deliver the crown ether derivative **482** (28%). Subsequently, compound **482** was treated with potassium hexacyanoferrate to get the desired cyclophane **483** via an intramolecular oxidative coupling (Scheme 89).

#### Intramolecular S_N_Ar reaction

**Scheme 90:**
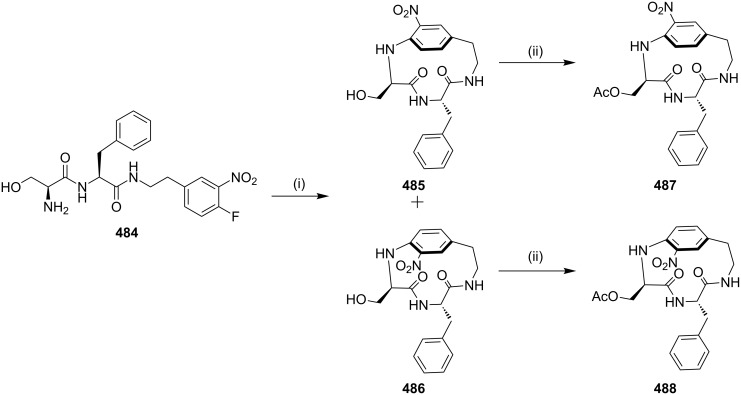
Synthesis of cyclopeptide via an intramolecular S_N_Ar reaction. Reagents and conditions: (i) TBAF, THF, MS 3 Å, rt, 68%; (ii) Ac_2_O, CH_2_Cl_2_, Et_3_N, DMAP, rt, 72%.

In 2002, Zhu and co-workers [254] have synthesized cyclopeptide alkaloids containing paracyclophane with a peptidic tether via an intramolecular S_N_Ar reaction. Compound **484** was subjected to a ring closure in THF with TBAF as a base to give a mixture of two isomers **485** and **486** (65%). Subsequent acetylation gave cyclophane derivatives **487** and **488** (Scheme 90).

#### Muscopyridine via C-zip ring enlargement

**Scheme 91:**
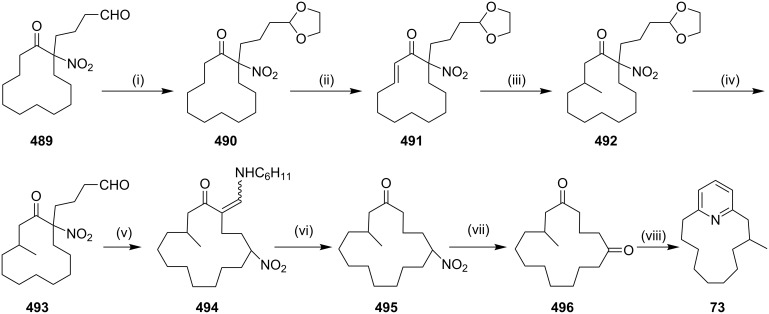
Synthesis of muscopyridine (**73**) via C-zip ring enlargement reaction. Reagents and conditions**:** (i) HO(CH_2_)_2_OH, TsOH, benzene, ∆; (ii) (a) LDA, PhSeBr, THF, −78 °C; (b) H_2_O_2_, AcOH; (iii) Me_2_CuLi, PhMe, −50 °C; (iv) TsOH, acetone, H_2_O; (v) C_5_H_11_NH_2_, EtOH, 23 °C; (vi) 10% HCl, EtOH; (vii) (a) MeOH, MeONa, TiCl_3_, NaOAc, (b) HCl, H_2_O; (viii) NH_2_OH·HCl, EtOH, 165 °C, 64%.

**Figure 13:**
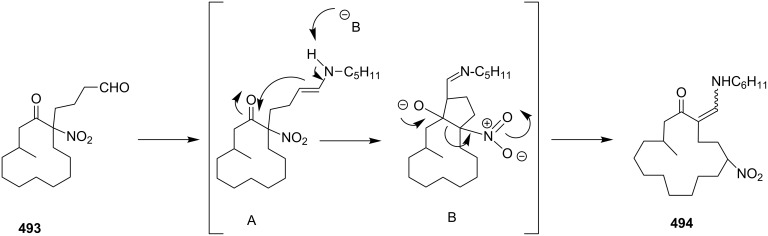
Mechanism of the formation of compound **494**.

Hadjabo and Hesse [255] have synthesized muscopyridine (**73**) via the C-zip ring enlargement reaction as a key step (Scheme 91). Aldehyde **489** was protected with ethylene glycol to generate the mono-acetal **490**. Then, enone **491** was afforded with lithium diisopropylamide (LDA) and PhSeBr/H_2_O_2_. The intramolecular conjugated addition of the enone system **491** in the presence of Me_2_CuLi gave a mixture of two diastereomers **492**. The deprotection of the ketal with TsOH furnished aldehyde **493**. A ring expansion involving an enamine reaction gave compound **494** (Figure 13), which was then hydrolyzed in 10% HCl to deliver **495**. Nitroderivative **495** was subjected to a modified Nef reaction with TiCl_3_ to deliver diketone **496**. Finally, diketone **496** was converted to a pyridine derivative with hydroxylamine hydrochloride to generate muscopyridine (**73**, Scheme 91).

#### Nicholas reaction

**Scheme 92:**
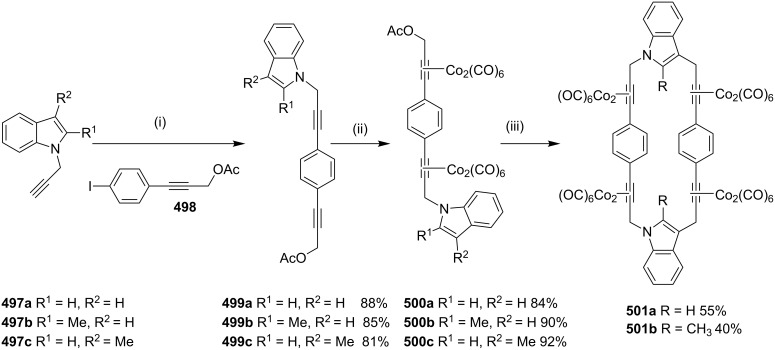
Synthesis of indolophanetetraynes **501a**,**b** using the Nicholas reaction as a key step. Reagents and conditions: (i) Pd(PPh_3_)_4_, CuI, iPr_2_NH, rt, 12 h; (ii) Co_2_(CO)_8_, Et_2_O, 0 °C, 3.5 h.; (iii) BF_3_·OEt_2_, 0 °C, 5 h.

Green and co-workers [256] have reported the synthesis of an indolophanetetrayne–cobalt complex by using the Nicholas reaction as a key step (Scheme 92). Sonogashira coupling of *N*-propargylindoles **497a–c** with iodoarylpropargyl acetate **498** gave N-functionalized indole precursors **499a–c** [257–258]. Both alkyne units of diynes **499a–c** can be converted to the corresponding cobalt complexes **500a–c** in the presence of an excess amount of Co_2_(CO)_8_. The protected complex **500a** was subjected to a cyclization reaction using BF_3_·OEt_2_ at room temperature to generate C-2-linked indolophanetetrayne **501a** (55%, Scheme 92).

#### Radical cyclization

**Scheme 93:**
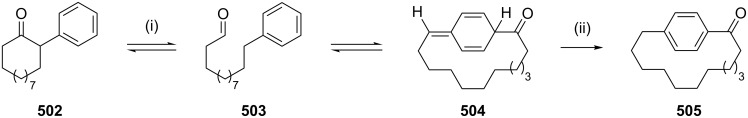
Synthesis of cyclophane via radical cyclization. Reagents and conditions: (i) cyclododecanone, phenyllithium 2 M, THF, –78 °C, *h*v, 2 h, 78%; (ii) 450 W mercury lamp, K_2_CrO_3_, 20–40 min.

In 1990, Turro and co-workers [259] have demonstrated a new methodology involving the photolysis of large α-phenylcycloalkanes by an intramolecular para coupling of the acylbenzyl biradical intermediate. Cyclododecanone **502** was subjected to photolysis to generate both α-cleavage and γ-hydrogen abstraction reaction to give compound **503**. The photochemical irradiation of the large-ring containing 2-phenylalkenones **504** produce cyclophane **505** as the major product (Scheme 93).

#### Ramberg–Bäcklund olefination reaction

**Scheme 94:**
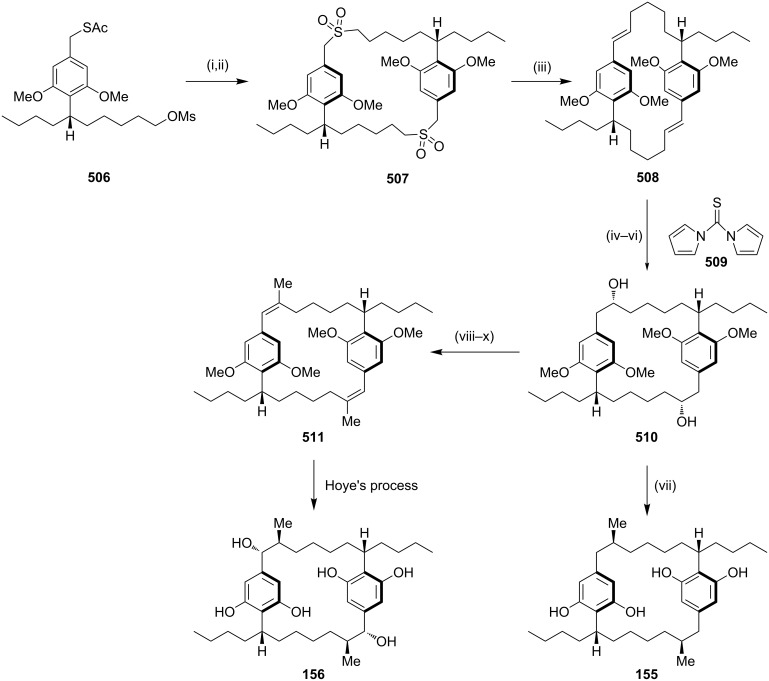
Synthesis of (−)-cylindrocyclophanes A (**156**) and (−)-cylindrocyclophanes F (**155**). Reagents and conditions: (i) NaOMe, MeOH, 23 °C, 36 h; (ii) (NH_4_)_6_Mo_7_O_24_·4H_2_O, H_2_O_2_, EtOH, 23 °C, 12 h, 51% over two steps; (iii) CF_2_Br_2_, KOH/Al_2_O_3_, CH_2_Cl_2_/*t*-BuOH 1:1, 0–23 °C, 2 h; then [Pd(CH_3_CN)_2_Cl_2_], 40 °C, 4 h, 70%; (iv) AD-mix-β, MeSO_2_NH_2_, *t*-BuOH/H_2_O 2:1, 23 °C, 12 h; (v) **509**, PhMe, 125 °C, 5 h; (vi) AIBN, *n-*Bu_3_SnH, PhMe, 100 °C, 1.5 h, 50% over three steps; (vii) MsCl, Et_3_N, CH_2_Cl_2_, 0 °C, 0.5 h; then AlMe_3_, 0 °C, 10 min; then BBr_3_, 23 °C, 5 h, 71% one pot; (viii) DMP, NaHCO_3_, CH_2_Cl_2_, 23 °C, 1 h; (ix) KHMDS, Comins reagent, THF, −78 °C, 1 h; (x) Fe(acac)_3_, MeMgBr, THF/NMP 20:1, 0 °C, 1 h, 74% over three steps.

In 2010 Nicolaou and co-workers [260] have reported the asymmetric synthesis of (−)-cylindrocyclophanes A and F (**156**, **155**) by employing the head-to-tail dimerization approach to this class of compounds, based on the Ramberg–Bäcklund olefination reaction. The monomeric bifunctional precursor **506** was dimerized to [7.7]paracyclophane by using NaOMe in MeOH at ambient temperature to generate macrocyclic bis(thioether). Macrocyclic bis(sulfone) **507** (51%) has been obtained by oxidation of bis(thioether) with H_2_O_2_ in the presence of (NH_4_)_6_Mo_7_O_24_·4H_2_O). Then, sulfone **507** on treatment with alumina-impregnated KOH (KOH/Al_2_O_3_) in the presence of CF_2_Br_2_ in CH_2_Cl_2_/*t*-BuOH 1:1 gave the bis(olefin) **508** (70%). The dihydroxylation of compound **508** with AD-mix-β (MeSO_2_NH_2_, *t*-BuOH/H_2_O 2:1 at ambient temperature) generated the tetrol which was selectively deoxygenated under Barton’s conditions to deliver diol **510**. The installation of the methyl group in **510** followed by a subsequent demethylation generated cylindrocyclophane F (**155**, 71%). Also, compound **510** was oxidized followed by enol triflate formation and Kumada-type coupling with MeMgBr to give bis(olefin) **511** (74%) which was further converted into cylindrocyclophane A (**156**) by using Hoye’s protocol [261] (Scheme 94).

#### Wittig reaction

**Scheme 95:**
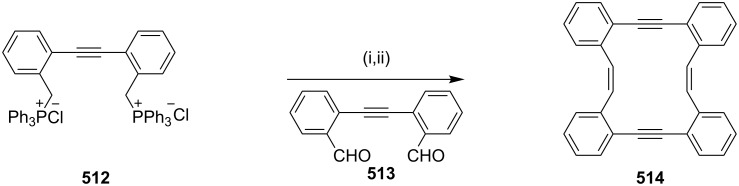
Cyclophane synthesis via Wittig reaction. Reagents and conditions: (i) LiOEt (2.1 equiv), THF, −78 °C to 0 °C, 0.5 h; (ii) **513** (1.1 equiv), THF, −78 °C to 0 °C, rt, 4 h.

π-Conjugated molecules are topologically interesting entities due to their structural and electronic properties. Various π-conjugated cyclophanes involving arylene–ethynylene or –ethenylene moieties have been reported in the literature. Otera and co-workers [262] have reported the synthesis of the magazine rack molecule **514** by using a Wittig reaction as a key step. In addition, these molecules were found to be quite fluxional even at low temperatures (Scheme 95).

**Figure 14:**
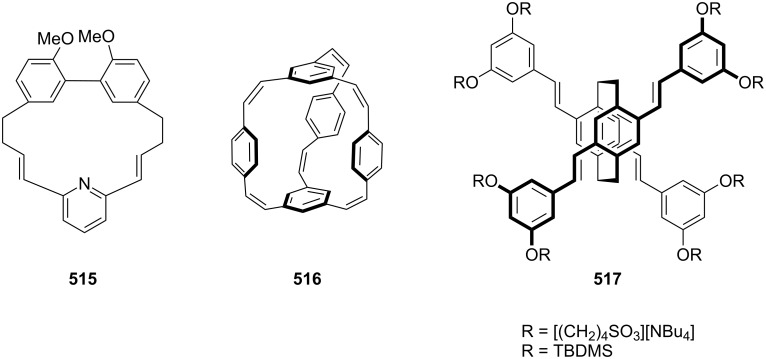
Representative examples of cyclophanes synthesized via Wittig reaction.

Over 40 different alkaloids were isolated from the Lythraceae family ranging from type A–E. Type C–E were reported previously, but Fujita and co-workers [263] reported the synthesis of type A alkaloid lythranidine for the first time. The key intermediate **515** was synthesized by using the McMurry reaction as a key step. For decades, caged compounds have been found to be useful targets to accommodate different ions. By a simple modification and the utilization of the flexibility of the crown ethers they can be used for the trapping of a variety of metal ions. Wennerström and co-workers [264] reported the synthesis of bicyclophane **516** by using a six-fold Wittig reaction. The use of conjugated polymers in chemical and biological sensors is well-known. However, water solubility poses limitations on the extensive use of these molecules. Bazan and co-workers [265] have reported the synthesis of the water-soluble oligomer dimers **517** based on paracyclophane with two chromophores in close proximity which results in a strong interchromophore delocalization and a decreased tendency toward aggregation as shown by light-scattering experiments (Figure 14).

#### Thermal isomerization of Dewar benzene

**Scheme 96:**
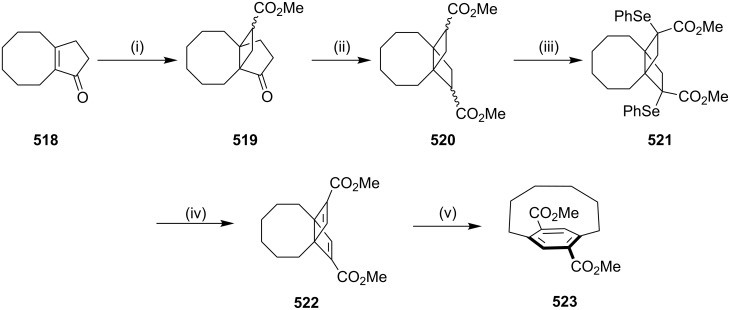
Synthesis of the [6]paracyclophane via isomerization of Dewar benzene. Reagents and conditions: (i) methyl acrylate, ether, 500 W, 3–5 h, 49%; (ii) HCO_2_Me, MeONa, TsN_3_, *h*v, MeOH, 2 h, 35%; (iii) LDA, THF, −78 °C, Ph_2_Se_2_, HMPA, 1 h, 32%; (iv) pyridine, CH_2_Cl_2_, H_2_O_2_, 1.5 h, 40 °C, 73%; (v) C_6_H_6_, 50 °C, 95 h, 90%.

In 1987, Tobe and co-workers [266] have explored different routes to assemble the [6]paracyclophane structure by utilizing thermal valence isomerization of Dewar benzene. The photocycloaddition of bicyclic enone **518** with methyl acrylate gave the head-to-tail endo product **519** (49%), which was subjected to ring contraction via (i) α-formylation (ii) diazo-transfer and (iii) Wolff photo rearrangement to generate propellane derivative **520** (35%). Phenylselenylation of **520** with an excess amount of LDA and diphenyl diselenide gave bis-selenide **521** (32%). Oxidation of **521** with hydrogen peroxide generated the Dewar benzene derivative **522** (73%). Finally, valence isomerization of propellane derivative **522** afforded [6]paracyclophane **523** (90%, Scheme 96).

## Conclusion

We have summarized the utility of various popular reactions related to cyclophane synthesis. In some instances, cyclophanes are formed in low yield and also with side products. We feel that this compilation will be beneficial to design better routes and to improve the existing routes to cyclophanes. We have included popular reactions which in our view have potential for further expansion. We have also included structures of interesting cyclophane derivatives without going into detailed schemes to keep the volume of information at a manageable level.
